# Assessment of the Impact of Scientific Reports Published by EFSA and GIS on Functional Foods Newly Placed on the Market in Poland

**DOI:** 10.3390/ijerph19074057

**Published:** 2022-03-29

**Authors:** Kacper Wróbel, Anna Justyna Milewska, Michał Marczak, Remigiusz Kozłowski

**Affiliations:** 1 Department of Management and Logistics in Healthcare, Medical University of Lodz, 90-131 Lodz, Poland; michal.marczak@umed.lodz.pl; 2 Department of Statistics and Medical Informatics, Medical University of Bialystok, 15-089 Bialystok, Poland; anna.milewska@umb.edu.pl; 3 Centre for Security Technologies in Logistics, Faculty of Management, University of Lodz, 90-237 Lodz, Poland; remigiusz.kozlowski@wz.uni.lodz.pl

**Keywords:** dietary supplements, food supplements, EFSA, GIS

## Abstract

Dietary supplements are health-promoting products. The legal categorization of dietary supplements as foods does not raise concerns, but a general understanding of how they work in the human body seems to deviate from the official definition. Thus, it is necessary to establish effective methods of market control related to dietary supplements. This research aims at assessing the impact of recommendations by various food safety authorities on ingredients used in newly registered products. It probes how the proportions of utilized product ingredients were modified after the European Food Safety Authority (EFSA) and Chief Sanitary Inspector in Poland (GIS) published their recommendations. Research data on the composition of products comes from the Polish national register of dietary supplements and covers the period from 2012 to 28 November 2021. Note that 103,102 products were analysed for the presence of thirty-seven ingredients, and the joinpoint regression method was applied to assess changing trends related to the use of ingredients. As our research points out, most often, changes in the trend appeared in product ingredients for which the European Food Safety Authority and Chief Sanitary Inspector in Poland issued the recommendation of having the safest level of consumption. However, these changes seem to emerge randomly and should not be unquestionably considered as the result of the published recommendations.

## 1. Introduction

Dietary supplements (DS) are included in the group of healthy products and, as foods they are supervised by the state sanitary inspection bodies. The decision to consume DS is often initiated by the consumers themselves, and it may be opposed [1]. Their legal status and social perception present various challenges in the areas of their effectiveness, safety, regulations, and clinical approach [2].

No uniform and standardized method of supervision over healthy products already placed on the market exists in the Member States of the European Union. For this reason, it is necessary to search for effective methods of market control [3,4]. This is especially important in terms of the emerging awareness concerning the adverse effects experienced by consumers of dietary supplements [5,6,7].

Although the legal categorization of dietary supplements as foods does not raise concerns, the consumers’—or rather, the patients’—understanding of their function seems to stray far from the official definition [8]. This is important since certain ingredients in dietary supplements can hardly be recognised as components of a normal diet, which supplements are intended to enhance. For instance, red yeast rice is one such ingredient that is promoted as a cholesterol-lowering remedy [9]. The perception of dietary supplements as being medical products rather than food formulations is demonstrated by the extended statutory definition published in 2006, which contains a provision that excludes from this group all products that have medicinal properties in accordance with the meaning of the pharmaceutical law [10]. This fact may point to the growing interpenetration of the pharmaceutical and the food markets, including dietary supplements. The forms of supplements include capsules, tablets, or lozenges, and evoke obvious associations with medicinal products, while the regulatory changes only emphasise the link between the two markets. 

In order to ensure effective monitoring associated with the safety of dietary supplements, all member states of the European Union can introduce registration models and requirements for products placed on the market. This solution is stipulated in Article 10 of Directive 2002/46/WE of the European Parliament and of the Council of 10 June 2002. on the approximation of the laws of the EU member states regarding food supplements. The directive allows the administrative bodies responsible for ensuring the safety of consumers in individual countries to require companies to inform them about placing dietary supplements on the market by submitting a template of the product’s label [11].

Despite the optional character of Art. 10 of the directive, most member states follow and set up monitoring systems/models for dietary supplements placed on the market. These systems can vary and possess different degrees of impact on the freedom to launch new products [3].

In the EU countries, it is possible to recognise four monitoring systems for dietary supplements on the market. They are characterised by various degrees of freedom related to implementing new products. The first and smallest group of EU member states includes countries that did not decide to introduce any notification system. Thus, entrepreneurs have the possibility to place healthy foods on the market freely, but they must meet the general and specific requirements of food laws. Obeying the law is verified during frequent official controls of food products [12,13,14,15,16,17,18]. However, the second, most popular, solution is a notification system that obliges food business operators to notify the food authorities of their intention to launch the sale associated with a particular dietary supplement. Nonetheless, approval or authorization by the institution in question is not required [19,20,21,22,23,24,25,26]. This system of notification on placing new healthy food products on the market also exists in Poland [27]. Note that selected EU states enlarge the above system or model by creating an additional obligation of paying administrative fees [28,29,30,31,32,33,34,35,36,37]. Moreover, a group of European markets can be further specified by requiring that new supplements and healthy food products are authorised by regulatory organizations, which review a formal application seeking approval to the market free of charge [38], as well as for a fee [39]. A detailed overview of the models of registration and authorization of healthy products is presented in the Table 1 below.

Let us emphasise the unique character of the solution implemented in Poland. The adopted system is one of four out of a total of 28 analysed, which presupposes public availability of the register together with the full qualitative list of ingredients used in the respective products.

It is also worth mentioning that, despite the statistical reporting carried out for dietary supplements in the area of the value of marketed production—unlike in the case of other typical food products—it is impossible to obtain reliable data on the volume and value of production and sale. This may be caused by the lack of a separate group, both according to the Combined Nomenclature (CN) and the Polish Classification of Goods and Services [40]. This means that the discussed register of products is the sole official source containing information on healthy products placed on the market in Poland.

The aim of this research is to assess the impact of recommendations published by food safety authorities in Poland and the European Union linked to the quality and maximum safe levels of ingredients found in dietary supplements and other healthy food products newly registered in Poland.

## 2. Materials and Methods

In the context of the model of monitoring of products placed on the market presented above, attention should be paid to state administration institutions and bodies whose task it is to shape the scientific basis for the supervision of the safety of dietary supplements. Numerous official and non-governmental structures exist that are engaged in the process of risk assessment of using specific ingredients of healthy foods; this study, however, focuses on two of them, with an attempt to identify changes that occurred in the register of healthy products in the area of ingredients that are the subject of scientific recommendations.

### 2.1. EFSA

European Food Safety Authority (EFSA) is an agency financed by the European Union which operates independently from European legislative and executive bodies such as the Commission, the Council, or the Parliament, as well as the Member States. The office was set up in 2002 in response to the series of food crises that occurred towards the end of the 1990s. At that time, the agency was to be a scientific body and a source of information concerning the threats connected to the food chain. The agency was legally established by the European Union on the basis of general food law, i.e., Regulation (EC) No 178/2002 of the European Parliament and of the Council of 28 January 2002 laying down the general principles and requirements of food law, establishing the European Food Safety Authority, and laying down procedures in matters of food safety. In most cases, EFSA undertakes actions that respond to applications for scientific advice submitted by the European Commission, the European Parliament, or the Member States. The agency also carries out scientific research undertaken on its own initiative. This takes place particularly in situations when new problems and threats in the food chain arise, as well as in order to update the existing methods of assessment of known risks.

Scientific activities performed as part of the activities of EFSA are mainly carried out within the framework of scientific panels and committees. Members of these structures are appointed through open competitions. EFSA may also issue assessments and opinions prepared by its own staff members. EFSA employees monitor and analyse information and data concerning biological threats, chemical impurities, food consumption, and emerging threats [41,42].

#### EFSA’s Scientific Opinions

The following nine scientific opinions prepared and published by European Food Safety Authority were selected for the study:

- Scientific opinion on bovine lactoferrin (published 24 May 2012)
  In its summary of the opinion, EFSA’s Panel on Nutrition, Novel Foods and Food Allergens concluded that lactoferrin does not raise concerns in the area of safety, provided that it is consumed in the amount indicated in the opinion [43];
- Scientific opinion on the evaluation of the safety in use of yohimbe (Pausinystalia yohimbe (K. Schum.) Pierre ex Beille) (published 24 July 2013)
  The opinion states that the Panel was unable to determine the daily intake norms of Pausinystalia yohimbe bark and its preparations, which did not raise concerns as to their adverse impact on health. An assessment of exposure to Pausinystalia yohimbe from dietary supplements was made that showed that the theoretical maximum daily intake may exceed the maximum approved daily dose of Pausinystalia yohimbe used as a medicinal product [44];
- Statement on the post-marketing monitoring of the use of lycopene (published 9 January 2015)
  The opinion states that on the basis of previous assessments of intake norms performed by EFSA, the data concerning sale, and the data concerning the placement of products on the market provided for the period from July 2009 to June 2012, the Panel concluded that consumption of naturally occurring lycopene and its use as food colour or a new food ingredient within the accepted use levels does not lead to ADI exceeding 0.5 mg/kg bw/day [45];
- Safety of cranberry extract powder as a novel food ingredient pursuant to Regulation (EC) No 258/97 (published 12 May 2017)
  The opinion indicates that considering the composition, the process of production, supply, and the history of use of cranberry fruit, the Panel considers that the consumption of its derivatives does not give cause for safety concerns. The Panel concludes that cranberry extract powder is a safe food ingredient for the proposed uses and use levels [46];
- Safety of dried aerial parts of Hoodia parviflora as a novel food pursuant to Regulation (EC) No 258/97 (published 19 October 2017)
  The opinion is a response to the application submitted by the entrepreneur, who proposed a dose of 15 mg/portion, while the available data shows the safe dose to be 9.4 mg/day. For this reason, a negative decision concerning the application was issued [47];
- Scientific opinion on the safety of green tea catechins (published 14 March 2018)
  On the basis of the available data on the potentially negative impact of green tea catechins on the liver, the EFSA Panel concluded that evidence from interventional clinical trials shows that consuming doses equal to or exceeding 800 mg of EGCG/day as a dietary supplement induces a statistically significant increase in transaminases in the serum of persons undergoing treatment compared to the control group [48];
- Safety of synthetic trans-resveratrol as a novel food pursuant to Regulation (EC) No 258/97 (published 12 January 2016)
  The opinion presents EFSA’s position, which indicates that synthetic trans-resveratrol is safe provided that the consumed doses do not exceed 150 mg/day, and in adult persons only [49];
- Scientific opinion on the safety of monacolins in red yeast rice (published 3 August 2018)
  On the basis of the available information, the opinion emphasises that while the Panel was not able to determine the level of consumption of monacolin sourced from RYR in diet, this does not raise concerns as regards adverse health effects in the general population and, in applicable cases, in sensitive subsets of the population [50];
- Safety of astaxanthin for its use as a novel food in food supplements (published 5 February 2020)
  The above opinion updates the recommendations concerning astaxanthin intake norms. Considering the updated assessment of exposure to astaxanthin in the basic diet (fish and crustaceans) coupled with the 8 mg from dietary supplements, the NDA panel concluded the following: (i) such total exposure to astaxanthin is safe for adults, (ii) adolescents from 14 to <18 years reach the ADI, and (iii) the ADI exceeds 28% in children aged 10 to <14 years and up to 524% in infants aged 4–6 months [51].

The publication date criterion was the main one that determined whether an opinion was qualified for the study. Only opinions issued in the years 2012–2020 that pertained to substances used as ingredients in dietary supplements were included (opinions concerning additives, novel foods (pursuant to regulation 2015/2283, extended uses opinions and chemical/synthetic compounds), as well as enzymes, and opinions concerning materials and products for contact with food were excluded).

### 2.2. GIS

The system of food safety supervision in Poland covers the whole food chain, in accordance with the “from field to fork” principle. The resort responsible for compliance with the regulations in the area of food of non-animal origin is the Ministry of Health, while animal products available on the market are the formal responsibility of the Ministry of Agriculture and Rural Development. The most important governmental bodies that control food safety are the Chief Sanitary Inspectorate (GIS) and the General Veterinary Inspectorate. Both these inspectorates operate on the basis of regulations specifying their competences; however, the large majority of tasks connected with supervision over the production and distribution of dietary supplements and other healthy food products remain the responsibility of GIS [52].

#### 2.2.1. The Sanitary and Epidemiological Council’s Team for Dietary Supplements

The Team for Dietary Supplements is the Chief Sanitary Inspectorate’s advisory and opinion-forming unit. The Council operates on the basis of Article 9, Item 2a of the State Sanitary Inspectorate Act of 14 March 1985 [53]. The Team functions within the framework of the Sanitary and Epidemiological Council.

The Team’s tasks include, e.g., content-related and scientific support for the Chief Sanitary Inspector. The range of responsibilities in this respect primarily involves matters related to products covered by the notification obligation, specified in Art. 30 Item 1 of the Act on Food and Nutrition Safety. The effect of the Team’s work is written opinions in the form of resolutions, compiled lists of plant ingredients with their maximum doses in dietary supplements, as well as the norms that determine the maximum doses of vitamins and other mineral ingredients in the recommended daily portions of dietary supplements, exceeding which they exhibit medicinal properties. In addition, the Team is responsible for the monitoring of interactions and adverse effects of dietary supplements [54,55].

##### Resolutions of the Team for Dietary Supplements

In the analysed period, the Team published 37 resolutions, which pertained to a total of 43 ingredients used in products that are subject to the obligation of notification. In order to assess their impact, resolutions issued before the year 2021 were considered. These pertained to 28 ingredients, i.e., aloe, ashwagandha, beta-alanine, boron, chromium, zinc, fluorine, phosphorus, isoflavones, iodine, caffeine, folic acid, pantothenic acid, magnesium, manganese, copper, white mulberry, niacin, vitamin A, vitamin B1, vitamin B12, vitamin B2, vitamin B6, vitamin C, vitamin D, vitamin E, vitamin K, and iron. A detailed list of all the selected resolutions, including their prevalence and publication dates, is presented in Table A1.

### 2.3. Data Sourced from GIS Register

Data for the period 2012–2019 was collected on 27 April 2021. Data for the year 2020 reflects the state as of 21 March 2021, whereas data for the year 2021 (1 January 2021–28 November 2021) was collected on 28 November 2021. The data available in the register also covers an earlier period starting from 2007; however, notifications made from the beginning of 2007 up to May 2010 do not have a daily date of notification attributed (by default all notifications from this period are visible in the register with the date of 1 January of the given year). Moreover, data for the year 2011 included in the register is incomplete as it covers 200 items, while the counter embedded in the register website indicates a value of over 4000 notifications. Due to these limitations and inconsistencies, the data included in this study covers the period from the beginning of 2012 to 28 November 2021.

A total of 132,906 notifications were qualified for the first phase of the study. After excluding the notifications made in the years 2007–2011, 121,867 notifications were included in further analysis. Close to 25% of this number (24.46%) are notifications made by a single entity “X”, active in the years 2018–2021. Notifications made by company “X” in subsequent years were the following: 9% of all the notifications in 2018; 23% in 2019; in the year 2020 the proportion was 57%, while in 2021 (up to and including 28 November), the notifications made by entity “X” comprised 50% of all the notifications. It should be emphasised that the discussed company offers the so-called personalised supplementation. What this means in practise is that each modification to the formula performed by a given customer requires a new, repeated registration of the product. Such a model of distribution translates to a large number of notifications, which—unlike in the case of the typical model—may not correlate with the prevalence of a given product on the market. In the most radical version, one notification may denote only one product physically placed on the market. Due to the aforementioned high numbers of notifications made by entity “X”, it was excluded from further study. Ultimately, 103,102 notifications were included in the analysis.

The character of individual scientific opinions and resolutions may be differentiated according to their intended function. The analysed documents included the following opinions: (i) those determining the maximum dose of vitamins or minerals; (ii) those determining additional qualitative requirements concerning the ingredients subject to the opinion; (iii) those confirming the safety of use in food products; (iv) those indicating an action that is characteristic for medicinal products or even toxicity.

### 2.4. Data Analysis

An analysis of the products from the register in terms of the presence of particular ingredients was carried out with the use of the Text Miner function in Statistica 13.1 software (StatSoft). Text mining is a method that enables you to extract structured data from unstructured or partially structured sources such as text databases [56]. Considering the ingredients that may customarily appear in the register under various names, their synonyms were also included, i.e., thiamine and vitamin B1. All items from the register (each notification) were analysed using Text Miner function in terms of the presence or absence of each of the analysed ingredient. The result of the analysis was coded in the 0–1 system; where 0 denoted absence of the ingredient, while 1—its presence. Occurrences of the respective ingredients were summed up into quarters.

Joinpoint regression software (Version 4.9.0.0. March 2021) was used for calculations and graphic presentation. The joinpoint regression programme is a statistical solution that enables to test whether an apparent change in trend is statistically significant. This method consists of fitting several linear (or log-linear) segments to data, connecting in joinpoints where a change in the trend occurs [57].

The joinpoint regression programme was used to fit weighted least-squares regression models to the percentage of the presence of the ingredient on the logarithmic scale. It was assumed that the error random variable in the model was homoscedastic. For a joinpoint software run, a minimum of two observed time points in the beginning and ending line segments (including the joinpoint) and a minimum of two observed timepoints in any middle line segment (including the two joinpoints) were allowed. A minimum of zero and a maximum of five joinpoints were searched for using the Grid search algorithm, the permutation test, and an overall alpha level of 0.05. QPC is quarter percent change, which shows the rate of change in the presence of the ingredient over time. When interpreting this parameter, it is said that the presence of the ingredient changes each quarter by a fixed percentage [58].

## 3. Results

Depending on the results of the performed analysis, the ingredients were divided into the following four groups.

### 3.1. No Joinpoints Identified

#### 3.1.1. Upward Trend: Ashwagandha, Astaxanthin, Lactoferrin, Lycopene, Trans-Resveratrol, White Mulberry

**Figure 1:**
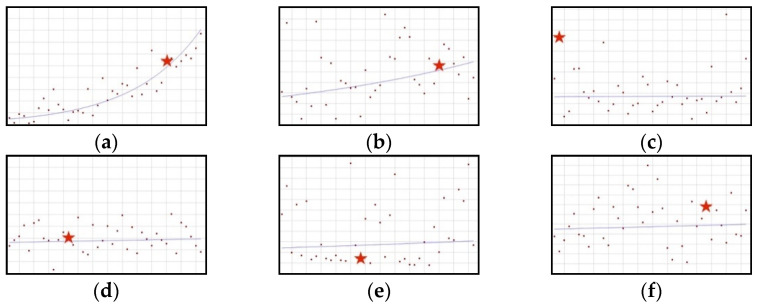
A schematic graph of the frequency of use of ingredients in the respective quarters in the period from the first quarter of 2012 to the fourth quarter of 2021. The star denotes the moment of publication of a scientific opinion or a resolution concerning the ingredient. The height of placement of the symbol reflects the level of use of the ingredient. (**a**): Ashwagandha (Figure A1); (**b**): Astaxanthin (Figure A2); (**c**): Lactoferrin (Figure A3); (**d**): Lycopene (Figure A4); (**e**): Trans-resveratrol (Figure A5); (**f**): White mulberry (Figure A6).

The proportion of all the ingredients in the group increases throughout the whole analysed period (Figure 1). This increase is statistically significant in the case of ashwagandha (QPC = 7.50) and astaxanthin (QPC = 2.09). No joinpoints can be observed after scientific opinions concerning the ingredients in question have been published.

#### 3.1.2. Downward Trend: Aloe, Caffeine, Chromium, ECGC, Hoodia, Niacin, Phosphorus, Red Yeast Rice, Vitamin B12

**Figure 2:**
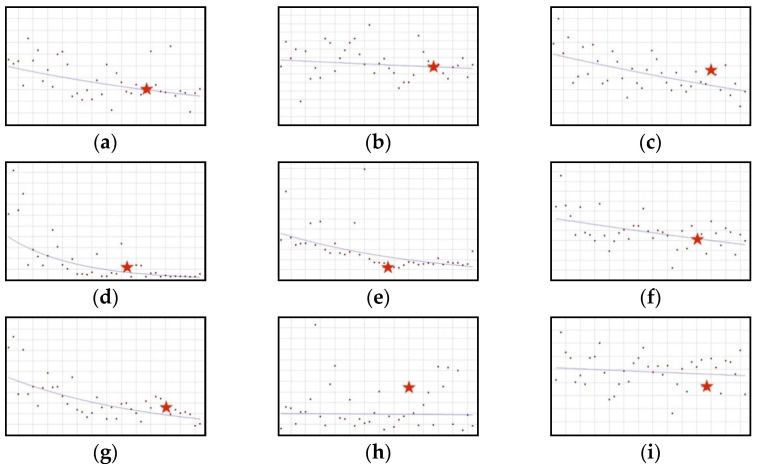
A schematic graph of the frequency of use of ingredients in the respective quarters in the period from the first quarter of 2012 to the fourth quarter of 2021. The star denotes the moment of publication of a scientific opinion or a resolution concerning the ingredient. The height of placement of the symbol reflects the level of use of the ingredient. (**a**): Aloe (Figure A7); (**b**): Caffeine (Figure A8); (**c**): Chromium (Figure A9); (**d**): ECGC (Figure A10); (**e**): Hoodia (Figure A11); (**f**): Niacin (Figure A12); (**g**): Phosphorus (Figure A13); (**h**): Red Yeast Rice (Figure A14); (**i**): Vitamin B12 (Figure A15).

The proportion of this group of ingredients in new healthy products in the whole tested period is decreasing (Figure 2); for six out of the nine ingredients, the decrease is statistically significant, i.e., aloe (QPC = −1.79), chromium (QPC = −0.96), ECGC (QPC = −6.96), hoodia (QPC = −3.18), niacin (QPC = −0.57), and phosphorus (QPC = −3.23). No joinpoints were present in the whole analysed period.

### 3.2. One Joinpoint Identified

#### 3.2.1. Upward-Downward Trend: Boron

**Figure 3:**
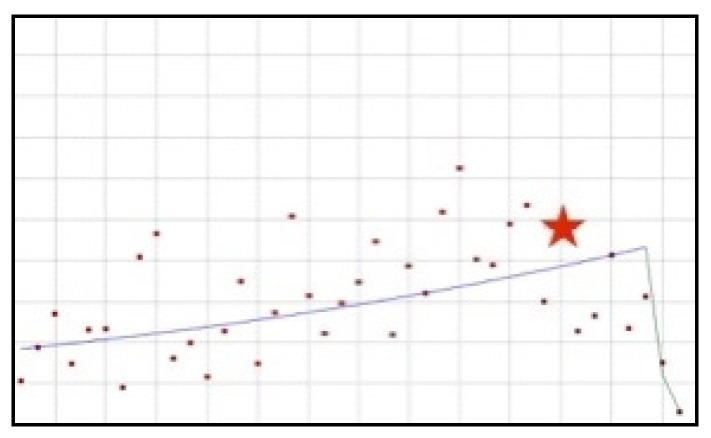
A schematic graph of the frequency of use of boron in the respective quarters in the period from the first quarter of 2012 to the fourth quarter of 2021. The star denotes the moment of publication of the resolution concerning the ingredient. The height of placement of the symbol reflects the level of use of the ingredient. Boron (Figure A16).

Using boron in newly registered healthy products in the time from the beginning of the analysed period to the second quarter of 2021 increased significantly (QPC = 2.32), while after this period a strong downward trend (QPC = −71.96) (Figure 3) occurred. The resolution that determines the maximum doses of boron in dietary supplements was published in the first quarter of 2020, so the change occurred five quarters after the quantitative norms of use were established.

#### 3.2.2. Downward-Upward Trend: Copper, Fluorine, Iodine, Iron, Isoflavones, Magnesium, Vitamin A, Vitamin B1, Vitamin B2, Vitamin B6, Vitamin C, Vitamin E

**Figure 4:**
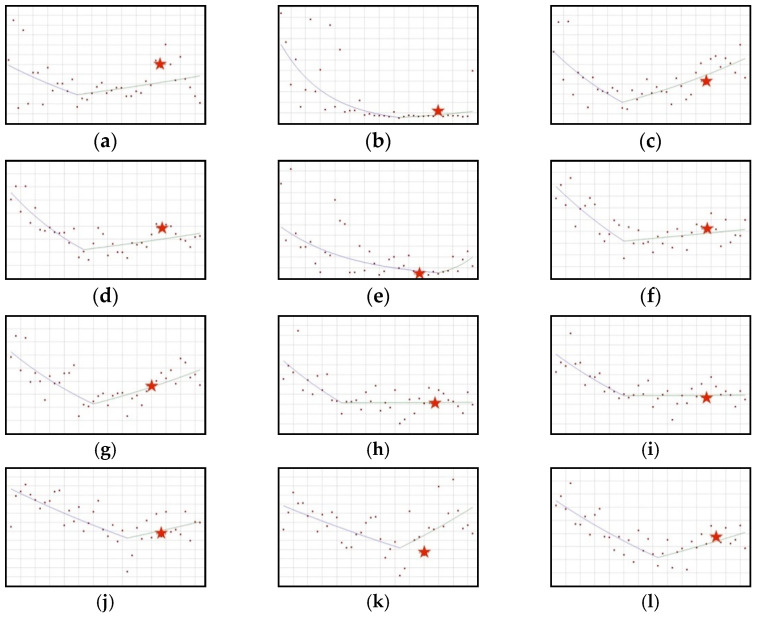
A schematic graph of the frequency of use of the ingredients in the respective quarters in the period from the first quarter of 2012 to the fourth quarter of 2021. The star denotes the moment of publication of a scientific opinion or a resolution concerning the ingredient. The height of placement of the symbol reflects the level of use of the ingredient. (**a**): Copper (Figure A17); (**b**): Fluorine (Figure A18); (**c**): Iodine (Figure A19); (**d**): Iron (Figure A20); (**e**): Isoflavones (Figure A21); (**f**): Magnesium (Figure A22); (**g**): Vitamin A (Figure A23); (**h**): Vitamin B1 (Figure A24); (**i**): Vitamin B2 (Figure A25); (**j**): Vitamin B6 (Figure A26); (**k**): Vitamin C (Figure A27); (**l**): Vitamin E (Figure A28).

All ingredients from this group recorded a decrease in use in new healthy products (Figure 4). In the case of all the ingredients except copper the decrease is statistically significant, with the following QPC values: fluorine (−10.19), iodine (−4.27), iron (−4.63), isoflavones (−6.63), magnesium (−2.87), vitamin A (−3.26), vitamin B1 (−3.15), vitamin B2 (−2.75), vitamin B6 (−1.19), vitamin C (−0.99), vitamin E (−1.83). GIS resolutions issued in relation to these ingredients were generally published in the period of increased use of the given raw material and, later, in relation to the identified joinpoints. An exception is isoflavones, in which case the resolution was published in the first quarter of 2019. A year later, the beginning of an upward trend in the use of isoflavones can be observed. Five out of the 11 ingredients whose use in new products significantly decreased showed the following statistically significant increases of use: iodine, QPC = 2.20; iron, QPC = 1.09; vitamin A, QPC = 1.84; vitamin C, QPC = 1.56; vitamin E, QPC = 1.06.

#### 3.2.3. Downward-Downward Trend: Pantothenic Acid

**Figure 5:**
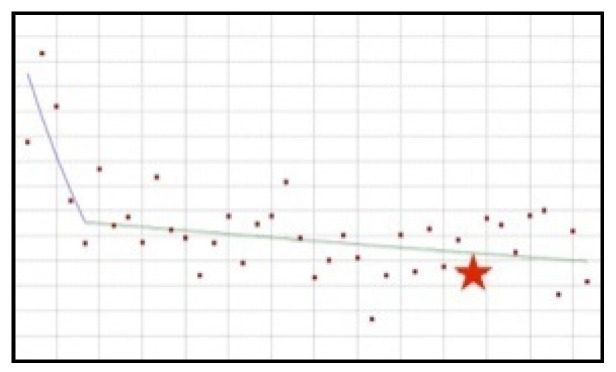
A schematic graph of the frequency of use of pantothenic acid in the respective quarters in the period from the first quarter of 2012 to the fourth quarter of 2021. The star denotes the moment of publication of the resolution concerning the ingredient. The height of placement of the symbol reflects the level of use of the ingredient. Pantothenic acid (Figure A29).

The use of pantothenic acid in the whole analysed period was decreasing (Figure 5), with a statistically significant decrease occurring in the time from the first quarter of 2013 up until the end of the analysed period, i.e., until the fourth quarter of 2021 (QPC = −0.46). The resolution concerning pantothenic acid was published in the fourth quarter of 2019, during the period of a constant decrease in the use of the ingredient.

### 3.3. Two Joinpoints Identified

#### 3.3.1. Downward-Upward-Downward Trend: Folic Acid, Manganese, Zinc

**Figure 6:**
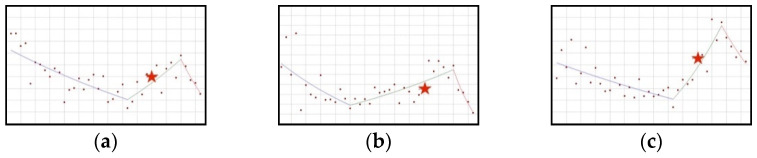
A schematic graph of the frequency of use of the ingredients in the respective quarters in the period from the first quarter of 2012 to the fourth quarter of 2021. The star denotes the moment of publication of a scientific opinion or a resolution concerning the ingredient. The height of placement of the symbol reflects the level of use of the ingredient. (**a**): Folic acid (Figure A30); (**b**): Manganese (Figure A31); (**c**): Zinc (Figure A32).

The use of folic acid and zinc significantly decreased in the period from the beginning of the study until the first quarter of 2018 (folic acid, QPC = −1.72; zinc, QPC = −1.33). Then, the use of both these ingredients significantly increased—for zinc, until the third quarter of 2020 (QPC = 5.87), while for folic acid, until the fourth quarter of 2020 (QPC = 3.26). Resolutions concerning the maximum levels of both these ingredients were published in the second quarter of 2019, i.e., approximately halfway through the period of increase (6 quarters after the occurrence of the joinpoint and 5/6 quarters before the end of the upward trend). In subsequent quarters, downward trends in the use of the discussed ingredients can be observed (Figure 6). The trends for manganese use are slightly different; its use was decreasing significantly from the third quarter of 2015 (QPC = −3.79), then it was increasing significantly for 21 quarters, until the end of 2020 (QPC = 2.31). At six quarters before the upward trend stopped (the second quarter of 2019), a resolution was published that determined the maximum levels of manganese in dietary supplements (Figure 6). Then, a statistically significant decrease in the use of the ingredient can be observed (QPC = −14.07).

#### 3.3.2. Downward-Downward Trend: Cranberry Derivatives

**Figure 7:**
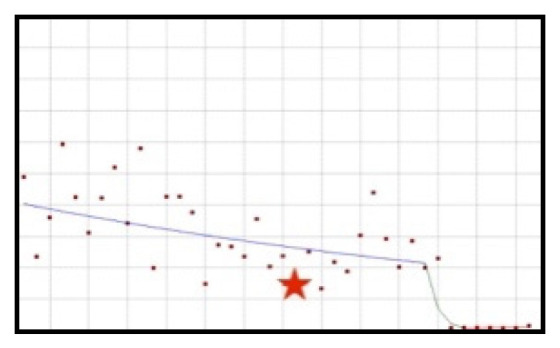
A schematic graph of the frequency of use of cranberry derivatives in the respective quarters in the period from the first quarter of 2012 to the fourth quarter of 2021. The star denotes the moment of publication of the resolution concerning the ingredient. The height of placement of the symbol reflects the level of use of the ingredient. Cranberry derivatives (Figure A33).

The use of cranberry preparations decreased throughout the whole analysed period (Figure 7). In the time from the beginning of the analysed period until the end of 2019, the decrease was statistically significant and characterised by slow dynamics (QPC = −2.00). In the period from the fourth quarter of 2019 to the third quarter of 2020, a sudden and statistically significant decrease in the use of cranberry derivatives was observed, while in the subsequent quarters only occasional cases of the use of this type of raw material occurred.

The scientific opinion concerning this ingredient was published in the second quarter of 2017, i.e., 13 quarters before the sudden drop in its use.

#### 3.3.3. Downward-Upward-Upward Trend: Vitamin K

**Figure 8:**
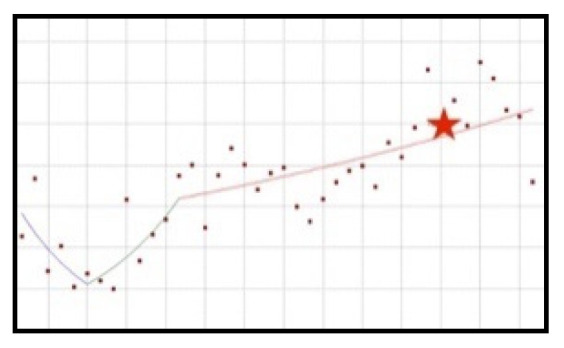
A schematic graph of the frequency of use of vitamin K in the respective quarters in the period from the first quarter of 2012 to the fourth quarter of 2021. The star denotes the moment of publication of the resolution concerning the ingredient. The height of placement of the symbol reflects the level of use of the ingredient. Vitamin K (Figure A34).

Throughout the analysed period, the trends of use of vitamin K were changing, with a significant decrease occurring in the period from the beginning of the analysed period until the second quarter of 2013 (QPC = −15.21), then increasing. The period when the increase takes place is divided into two phases (Figure 8). The first one lasts from the second quarter of 2013 until the first quarter of 2015, when the increase is dynamic (QPC = 14.66); the second one lasts until the end of the analysed period, with a smooth but statistically significant increase, for which the QPC value was 1.85. The resolution concerning vitamin K was published in the first quarter of 2020.

### 3.4. Three or More Joinpoints Identified

#### 3.4.1. Increase-Decrease-Increase-Decrease Trend: Yohimbine, Beta-Alanine

**Figure 9:**
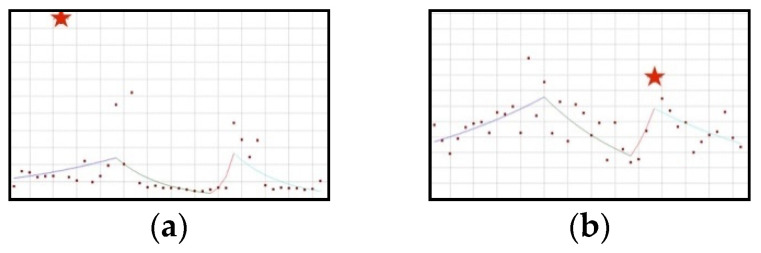
A schematic graph of the frequency of use of the ingredients in the respective quarters in the period from the first quarter of 2012 to the fourth quarter of 2021. The star denotes the moment of publication of a scientific opinion or a resolution concerning the ingredient. The height of placement of the symbol reflects the level of use of the ingredient. (**a**): Yohimbine (Figure A35); (**b**): Beta-alanine (Figure A36).

These ingredients are characterised by similar trends (Figure 9); however, in relation to each of them, scientific opinions and resolutions were published at various points and their characters were different.

The use of yohimbine in new healthy products was increasing from the beginning of the analysed period until the second quarter of 2015. Approximately halfway through this period, an EFSA scientific opinion was published (third quarter of 2013). 7 quarters later, after the upwards trend stopped, a statistically significant decrease of the frequency of use of cranberry preparations can be noted (QPC = −15.57), which lasted for a further 12 quarters. After this period, a dynamic, albeit short-lasting, 3-month increase in the use of the ingredient is visible, after which a statistically significant decrease occurs, from the first quarter of 2019 until the end of the analysed period (QPC = −15.02).

Beta-alanine is characterised by a distribution of popularity similar to yohimbine. From the beginning of the analysed period, the use of this ingredient was increasing significantly (QPC = 4.23) up to and including the third quarter of 2015, after which a significant decrease was noted (QPC = −7.55) which lasted another 11 quarters. In the period from the second quarter of 2018, an upward trend can be observed lasting until the first quarter of 2019. In the same quarter, a resolution was published that established the norms of use of beta-alanine in dietary supplements. From that moment until the end of the analysed period, a statistically significant decrease of use was observed (QPC = −4.39).

#### 3.4.2. Downward-Upward-Upward Trend: Vitamin D

**Figure 10:**
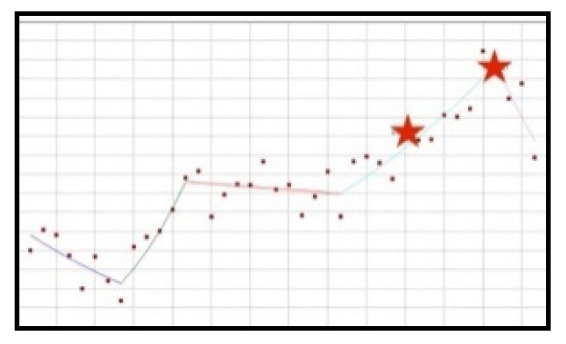
A schematic graph of the frequency of use of vitamin D in the respective quarters in the period from the first quarter of 2012 to the fourth quarter of 2021. The star denotes the moment of publication of the resolution concerning the ingredient. The height of placement of the symbol reflects the level of use of the ingredient. Vitamin D (Figure A37).

The use of vitamin D exhibited a downward trend only in the initial period of the study (until the fourth quarter of 2013). In the subsequent quarters, its use in new products increased considerably, with a sudden and dynamic increase until the first quarter of 2015 (QPC = 14.99). For the first quarter of 2015, for the next 3 years, a slight downward trend was observed; however, from the first quarter of 2018, for the next 3 years, it increased significantly (QPC = 4.40). Two resolutions of the Dietary Supplements Team were published in this period-in the second quarter of 2019 and at the end of the period of increase, i.e., in the first quarter of 2021 (Figure 10). From the moment of the publication of the latter resolution, the beginning of a downward trend in the use of vitamin D can be observed.

## 4. Discussion

As far as the assessment of the impact of the selected scientific opinions on the popularity of the used ingredients (that are their subjects) in new healthy products is concerned, it seems that both these areas (scientific opinions and used ingredients) are mutually independent. The ingredients, with significantly changed usage records in the analysed period, had joinpoints occurring at the following various times: from the same quarters in which the scientific opinions were issued, through three quarters after the opinion, to joinpoints occurring several quarters later after the publication. Considering the character of the published recommendations, it was observed that in the largest number of cases (28) are the scientific opinions and resolutions determined the maximum doses permitted in dietary supplements. This group included as many as 18 ingredients, in which a history of using a joinpoint was identified. Note that in eight cases, the joinpoint occurred earlier than 10 quarters after the announcement recommendation (including four cases with a joinpoint occurring up to five quarters after the recommendation and four cases with a joinpoint in the period from the fifth to the tenth quarter). In all the above-mentioned situations, except for isoflavones, a visible decrease in the trend took place in the joinpoint that occurred after the publication of the resolution determining the maximum permitted doses.

In turn, among the five published recommendations establishing additional qualitative requirements linked to the ingredients, three of them also determined their maximum doses. In this group, only two ingredients had joinpoints in their analysed history of use, but only one of them occurred more than 10 quarters later after the scientific opinion was published. It was related to cranberry derivatives.

Regarding the scientific opinions confirming the safety of using specific ingredients in food products and healthy foods, it was observed that four out of six cases in this group were characterised by an upward trend of use, and two cases had a downward trend. Interestingly, in the case of three scientific opinions questioning the safety of having certain ingredients, two ingredients were characterised by a consistently decreasing trend of use, while one, related to yohimbine derivatives, changed the trend from upward to downward in a period of time shorter than 10 quarters from the moment the opinion was published.

In the case of three ingredients (boron, isoflavones, and cranberry derivatives), the published recommendation resulted in an increase in the use of the ingredient. After the scientific opinion was published, the average time of the occurrence of a joinpoint appeared to be slightly longer than 5 quarters. At the same time, after an EFSA scientific opinion was published, which stressed that it is impossible to determine a safe level of yohimbine in foods, a decrease in the use of this ingredient can be observed, excluding a short period of growth from 2018 to 2019.

Furthermore, beta-alanine and vitamin D (the second resolution) present singular cases in which the publication of a GIS resolution and the decrease in the use of the ingredient occurred within a single quarter.

Looking at the other ingredients that possessed changeable trends of use in new products during the analysed period of time and had a single joinpoint occurred, it is possible to recognise that resolutions were published only after the joinpoint occurred. This situation seemed to take place during the upward trend. Nevertheless, in the case of pantothenic acid, it exceptionally happened during the downward trend. On average, the above occurred 14 quarters from the moment of trend change as follows: the fastest (shorter than 10 quarters) was in the case of vitamin C, vitamin B6, and fluorine. A similar course of action related to the publication of GIS resolutions was also observed in the cases of folic acid, zinc, and manganese. Resolutions establishing the maximum levels of use of the other vitamins and minerals covered by this study were published more than 10 quarters after the beginning of the upward trend.

To interpret the obtained results comprehensively, several factors should be taken into consideration. First of all, it is difficult to conclusively determine what time, after a recommendation is issued, should be considered the beginning of a trend change. Limited literature data on this topic describes extreme behaviour exhibited by some food industry companies in response to the need to adhere to sector recommendations. For instance, literature points out cases when a significant reduction in the calorie content of drinks delivered to schools was achieved within a two-year period since the publication of a sector manual [59], but at the same time, it accentuates other cases in which, despite declarations made by the producers concerning adequate product marketing aimed at children, only slightly over 10% of the assessed products complied with the criteria specified by the WHO [60]. Moreover, as the case of titanium dioxide shows, which safety in food products cannot be confirmed [61], the time from the moment the scientific opinion is issued [62] until the eventual date of its withdrawal from the food market can be as long as five quarters [63].

The amount of time needed to produce a dietary supplement should also be taken into consideration as it largely depends on the availability of ingredients. In extreme cases, the availability of certain ingredients may depend on transport from Asia and may take several months [64]. This situation especially applies to derivatives of plant-based ingredients native to such regions. This includes the following: derivatives of reed yeast rice, ECGC, ashwagandha, and white mulberry [65]. In all the cases mentioned, official recommendations dictated additional quality requirements. Thus, even the precise adjustment of ingredient levels may not be sufficient to fulfil standards set by the recommendation. In some cases, the ingredients should be changed. This necessity may result in a prolonged time of market adaptation to the new norms. However, adaptation marketing strategies seem to be underestimated by officially published recommendations.

When examining changes in the use of cranberry derivatives, it should be stated that a certain limitation of the analysis was caused by linguistics. For instance, this insufficiency was visible in the area of identification of products containing cranberry derivatives. It should be emphasised that the EFSA recommendation concerning the above ingredient mainly pointed out “cranberry extract powder”. However, after considering specific characteristics of Polish grammar, all items in the register that contained the term “cranberry” (cranberry in the genitive case) were qualified for the study. This may mean that products that did not contain “cranberry extract powder” but also, for example, “cranberry juice” or “cranberry fruit” were also analysed. The modification of the method of registration of healthy products introduced in February 2020 should also be mentioned here. This change consisted of blocking the subjects that make the notification from freely entering the names of ingredients; instead, they were to be selected from a drop-down list. Despite the fact that the possibility of using “cranberry extract powder” is a direct result of the specific provisions of the food law in force in the European Union (the so-called novel food list), this particular ingredient is not available on the selection list. The effect of this is the absence in the register of products that include “cranberry” in the genitive in their list of ingredients since February 2020. The registering entity may choose from the list “cranberry” in the nominative only. Due to this state of affairs and the technical limitations, all the conclusions concerning the impact of the EFSA opinion on the placement of products containing “cranberry extract powder” on the market should be qualified as uncertain.

The phenomenon of the increase in the use of a given ingredient after the publication of a scientific opinion also requires a critical assessment. In this context, the aspect of the placement of unsold stock on the market—after the scientific or legal realities have changed—cannot be omitted. One of the phenomena observed after limitations in the area of the maximum upper doses of certain minerals and vitamins have been published is the change in labelling of the product in the area of the recommended daily intake. In practise, the determination of the maximum level of vitamin D in a daily dose of a dietary supplement at 2000 I.U. may lead to the appearance of products on the market that would recommend the consumption of a tablet. An example that illustrates this phenomenon very well is a dietary supplement containing 4000 I.U. of vitamin D in a single tablet, which is then registered again after new requirements in the area are introduced but with a dose of tablet, so that it would meet the norms of supply of the ingredient in question. Market practises of this kind may result in an increase in the total number of new notifications for registration. This may explain the constant upward trends in the use of certain ingredients.

On the other hand, it would also be risky to conclude that the response time of opinion-forming bodies to a theoretically increasing use of a given ingredient is related to its safety for consumers. For example, vitamin C belongs to the group of vitamins with a low risk of exceeding the upper level (UL), whereas manganese and copper belong to the group with a relatively high risk of overdose [66]. Despite this, the resolutions that regulate the use of the two latter minerals appeared later (in relation to the beginning of the upward trend) than in the case of vitamin C. This leads to the side conclusion that categorization or any other attempt at ordering the ingredients according to a criterion based on the date of publication of the opinion/resolution would be unjustified in the light of the analysed data.

The matter of the increasing supply of certain groups of ingredients is also not without significance. The authors’ own research shows that the increasing trends in the use of, e.g., zinc, vitamin C, and vitamin D correlated with the subsequent waves of the COVID pandemics [67]. The presence of this type of seasonal increase in popularity and demand for selected healthy products may make it difficult to observe the real impact of an opinion on the producers’ responses to the changes they imply.

It is important to note that the European Commission, on a request made by the Directorate-General for Health and Food Safety (DG SANTE), has undertaken the initiative to determine the maximum levels of vitamins and minerals added to food and dietary supplements in the form of a future regulatory resolution. In this area, public consultation in this area is planned during the second quarter of 2023, and the approximate time for accepting this regulation by the Commission is envisioned as the first quarter of 2024.

## 5. Conclusions

As a result of the analyses, the following relationships were formulated:

1. In most cases, publications of EFSA’s scientific opinions and GIS resolutions do not significantly change the trends of popularity or use of the ingredients that are the subject of the recommendations in newly registered products;
2. Changes in trends, understood as joinpoints, appearing chronologically after the publication of a scientific opinion/resolution, seem too removed in time to conclude, on the basis of the collected data, which they are the direct result of GIS and EFSA actions. It can be assumed that those recommendations that determine the maximum permitted levels of ingredients have potentially the strongest impact on the behaviour of producers;
3. Further studies are required to assess changes in the levels of particular ingredients. In addition, they may identify potential practises aimed at bypassing a set of norms established by opinion-forming bodies. In order to determine the safest/highest levels of vitamins and minerals in dietary products, this aspect is particularly important as it allows for the creation of proper food regulations;
4. A public register of dietary supplements may be an effective tool to support the non-official control of the market known as its self-control. However, any register of supplements can perform its function if and only if there is research, including data on doses of active ingredients in dietary products.

## Figures and Tables

**Table 1:** List of monitoring systems of dietary supplements placed on the marked for the first time in selected Member States of the European Union and EEA, and in Great Britain as of June 2021.

Model of Registration	State *
Free placement on the market	Austria, The Netherlands, Sweden, Great Britain, Norway, Switzerland, Slovenia
Notification free of charge; authorization not required	Denmark ^(3)^, Estonia ^(1)^, Poland ^(2)^, France ^(3)^, Germany, Ireland, Luxembourg, Portugal, Spain
Paid notification; authorization not required	Croatia (1500 HRK), Cyprus (50 EUR), Finland, Greece (300–600 EUR), Hungary (50,000 HUF) ^(1)^, Italy (160.20 EUR) ^(1)^, Latvia (125–380 EUR) ^(3)^, Lithuania (98–156 EUR) ^(1)^, Malta (10 EUR), Slovakia (50 EUR) ^(1)^
Notification free of charge; authorization required	Bulgaria ^(1)^
Paid notification; authorization required	Belgium (200 EUR) ^(1)^

^(1)^ register publicly available, without qualitative composition of ingredients; ^(2)^ register publicly available, with qualitative composition of ingredients; ^(3)^ register publicly available, with qualitative composition of ingredients, but with certain limitations. No digit in superscript means that the register is not publicly available. * in brackets, the amount of the administrative fee is given, if applicable.

**Figure A1:**
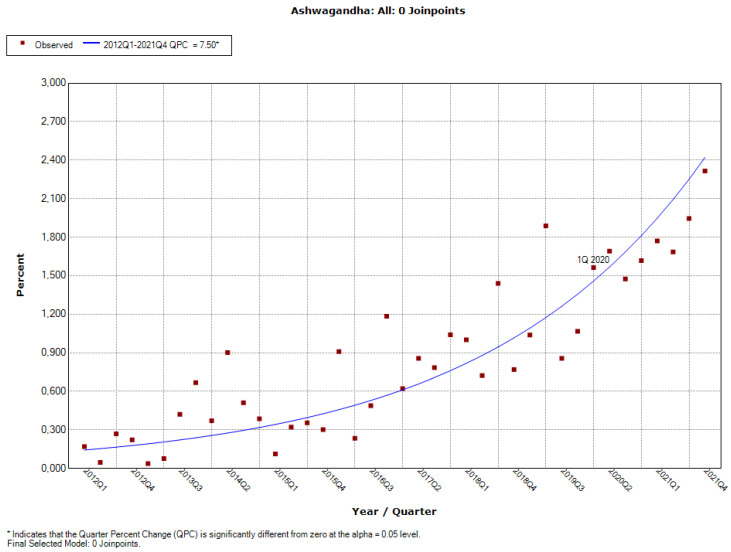
Percentage of products containing ashwagandha in individual quarters in the period from 2012 to 28 November 2021. Joinpoints mark trend turning points. The date above the observation indicates the moment of publication of the scientific opinion/resolution.

**Figure A2:**
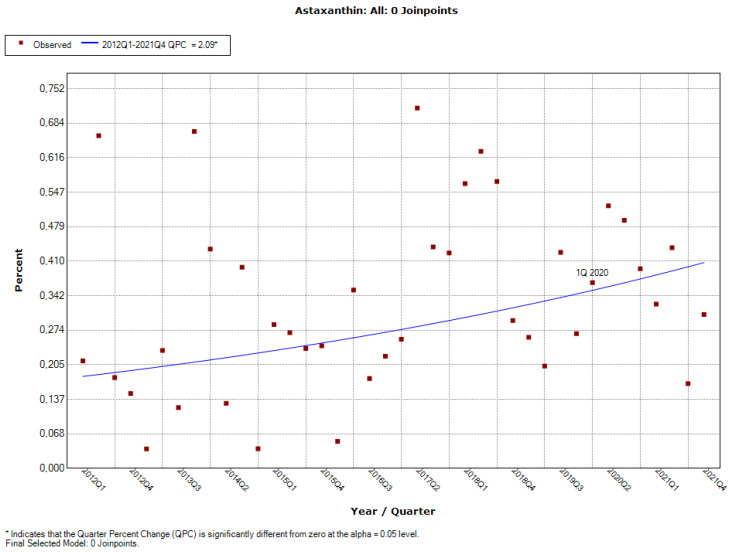
Percentage of products containing astaxanthin in individual quarters in the period from 2012 to 28 November 2021. Joinpoints mark trend turning points. The date above the observation indicates the moment of publication of the scientific opinion/resolution.

**Figure A3:**
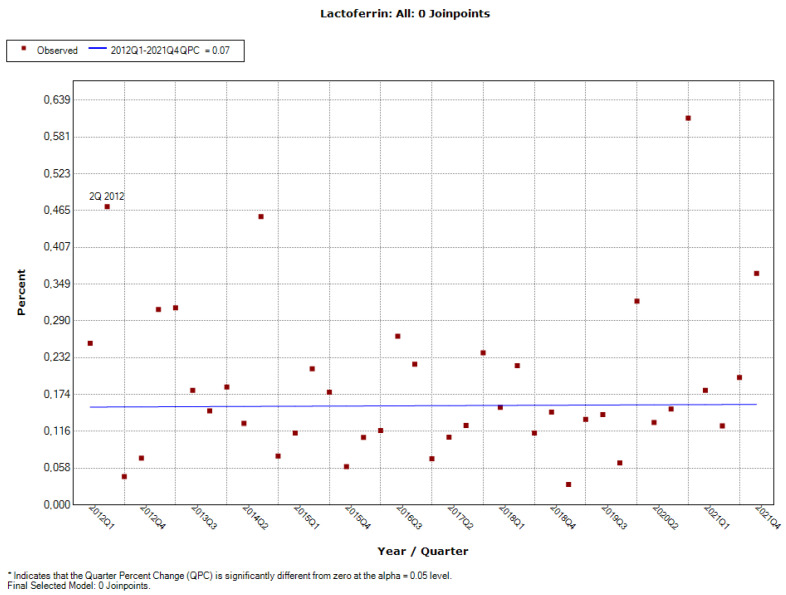
Percentage of products containing lactoferrin in individual quarters in the period from 2012 to 28 November 2021. Joinpoints mark trend turning points. The date above the observation indicates the moment of publication of the scientific opinion/resolution.

**Figure A4:**
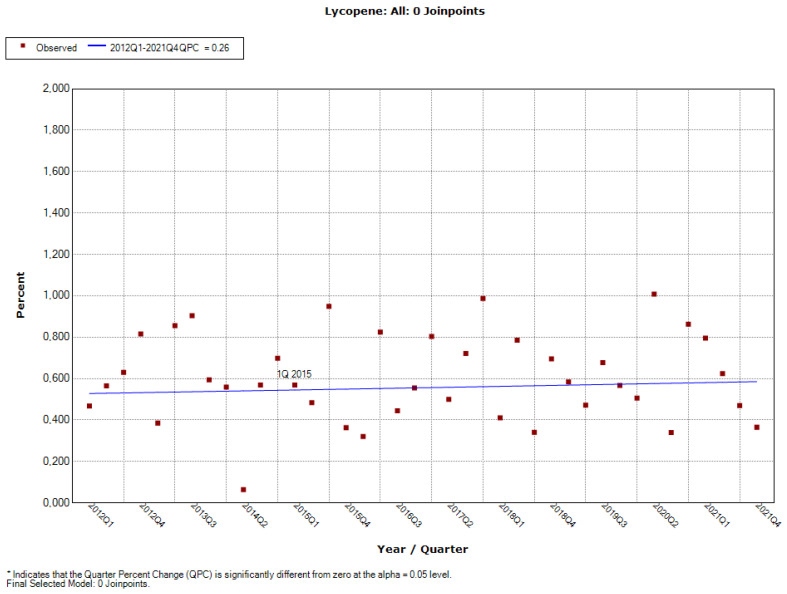
Percentage of products containing lycopene in individual quarters in the period from 2012 to 28 November 2021. Joinpoints mark trend turning points. The date above the observation indicates the moment of publication of the scientific opinion/resolution.

**Figure A5:**
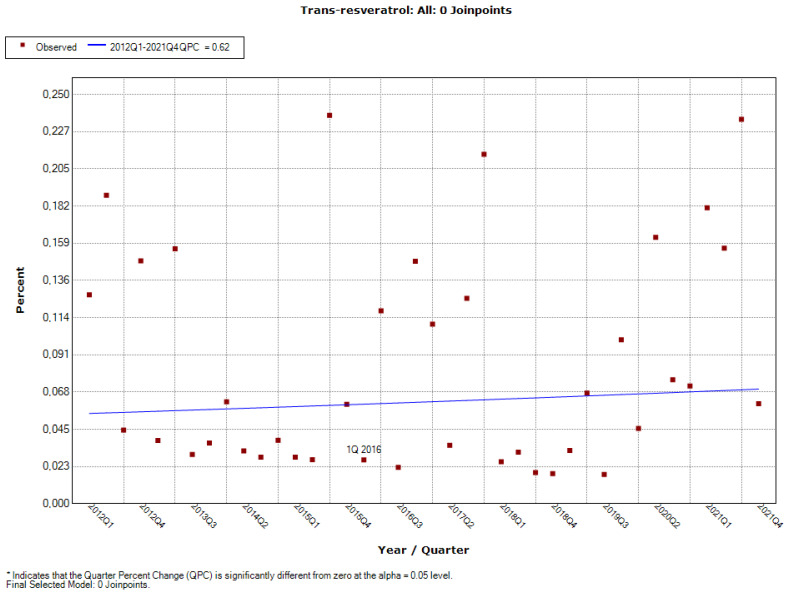
Percentage of products containing trans-resveratrol in individual quarters in the period from 2012 to 28 November 2021. Joinpoints mark trend turning points. The date above the observation indicates the moment of publication of the scientific opinion/resolution.

**Figure A6:**
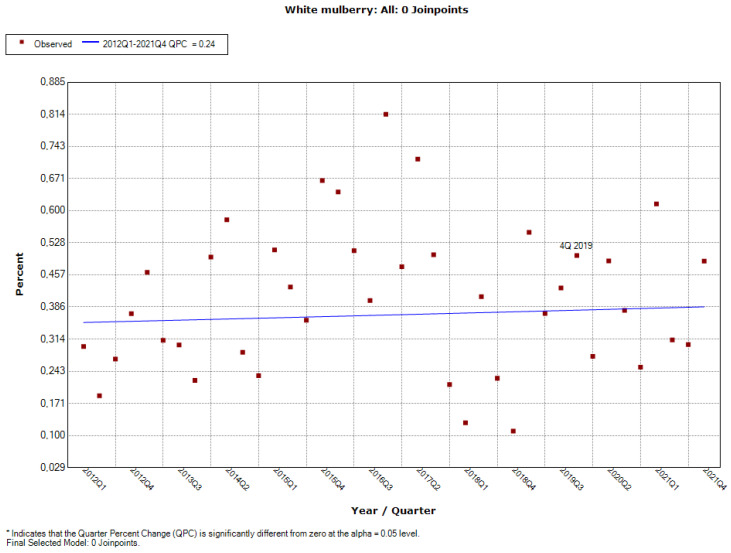
Percentage of products containing white mulberry in individual quarters in the period from 2012 to 28 November 2021. Joinpoints mark trend turning points. The date above the observation indicates the moment of publication of the scientific opinion/resolution.

**Figure A7:**
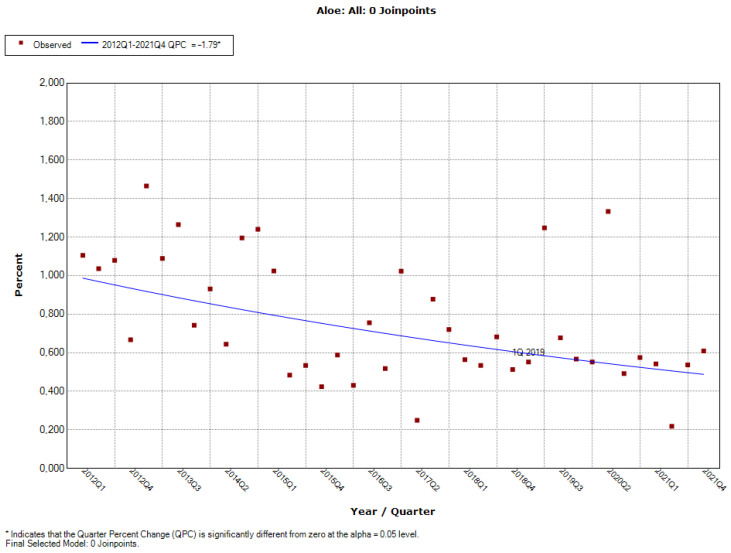
Percentage of products containing aloe in individual quarters in the period from 2012 to 28 November 2021. Joinpoints mark trend turning points. The date above the observation indicates the moment of publication of the scientific opinion/resolution.

**Figure A8:**
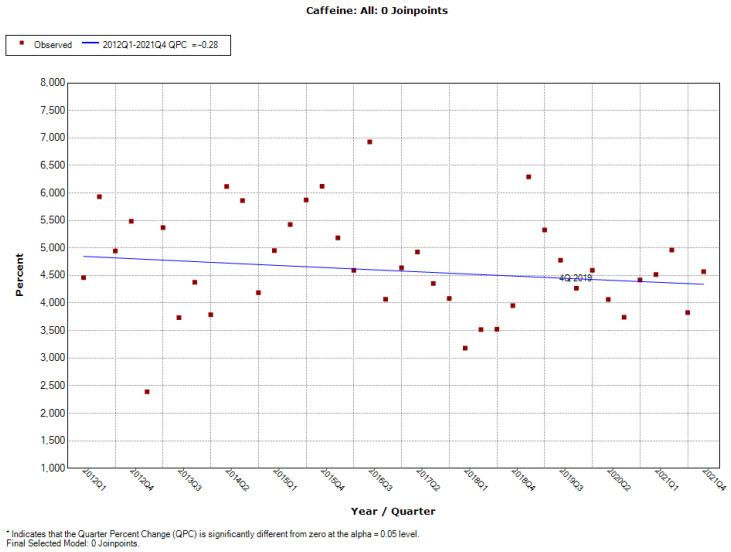
Percentage of products containing caffeine in individual quarters in the period from 2012 to 28 November 2021. Joinpoints mark trend turning points. The date above the observation indicates the moment of publication of the scientific opinion/resolution.

**Figure A9:**
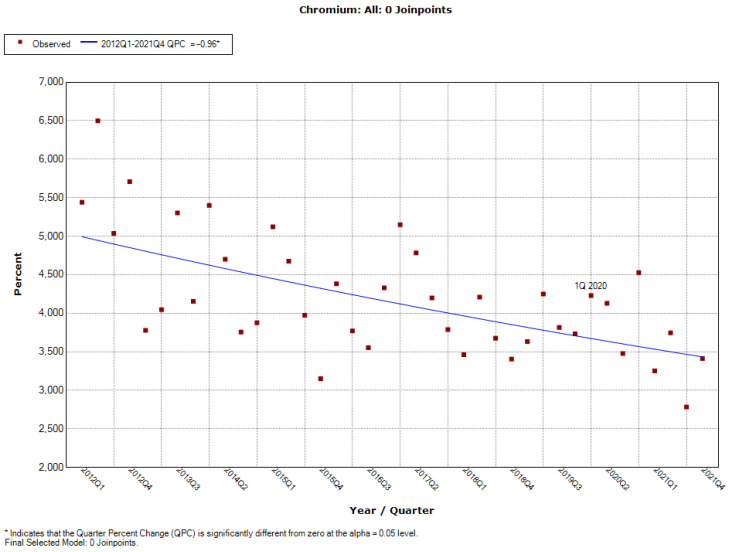
Percentage of products containing chromium in individual quarters in the period from 2012 to 28 November 2021. Joinpoints mark trend turning points. The date above the observation indicates the moment of publication of the scientific opinion/resolution.

**Figure A10:**
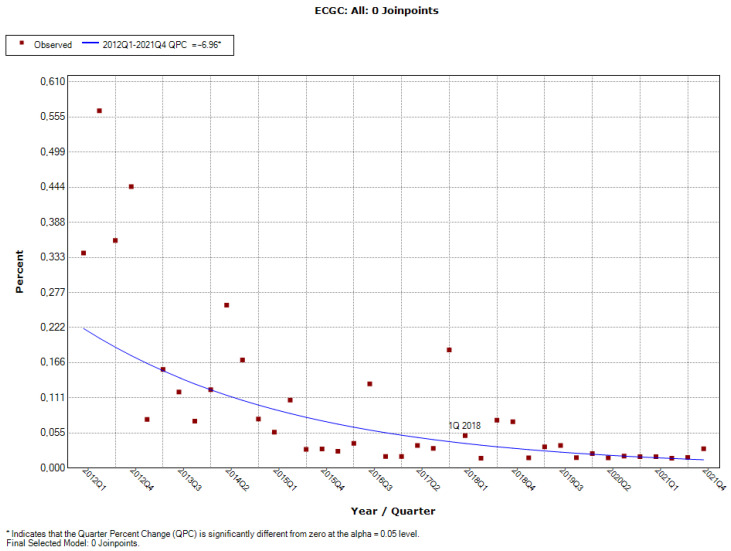
Percentage of products containing ECGC in individual quarters in the period from 2012 to 28 November 2021. Joinpoints mark trend turning points. The date above the observation indicates the moment of publication of the scientific opinion/resolution.

**Figure A11:**
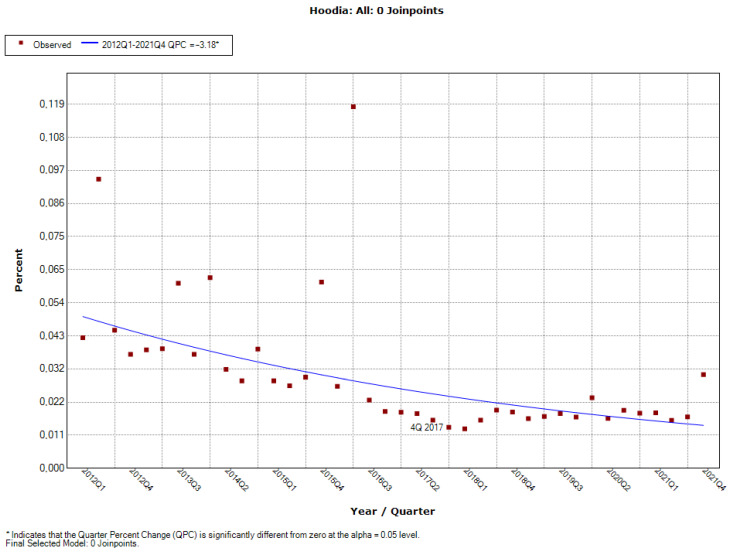
Percentage of products containing hoodia in individual quarters in the period from 2012 to 28 November 2021. Joinpoints mark trend turning points. The date above the observation indicates the moment of publication of the scientific opinion/resolution.

**Figure A12:**
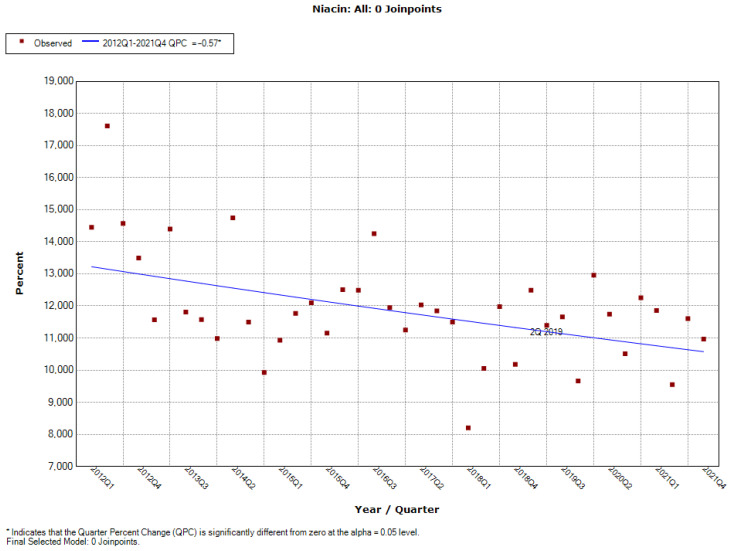
Percentage of products containing niacin in individual quarters in the period from 2012 to 28 November 2021. Joinpoints mark trend turning points. The date above the observation indicates the moment of publication of the scientific opinion/resolution.

**Figure A13:**
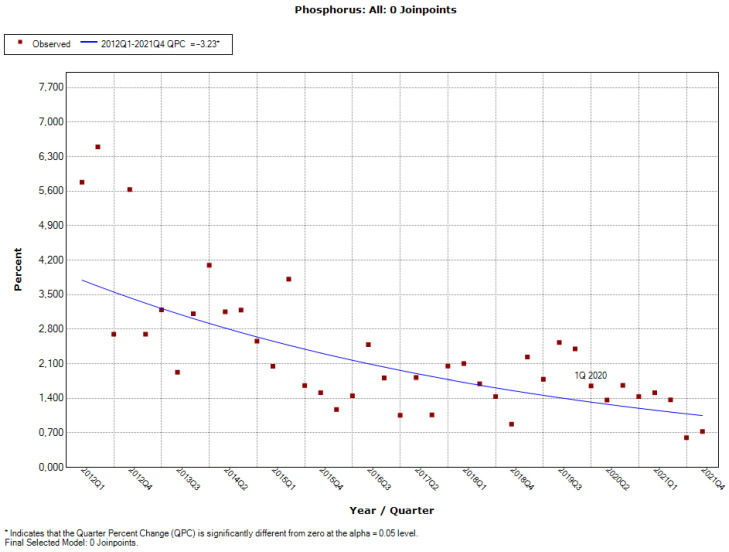
Percentage of products containing phosphorus in individual quarters in the period from 2012 to 28 November 2021. Joinpoints mark trend turning points. The date above the observation indicates the moment of publication of the scientific opinion/resolution.

**Figure A14:**
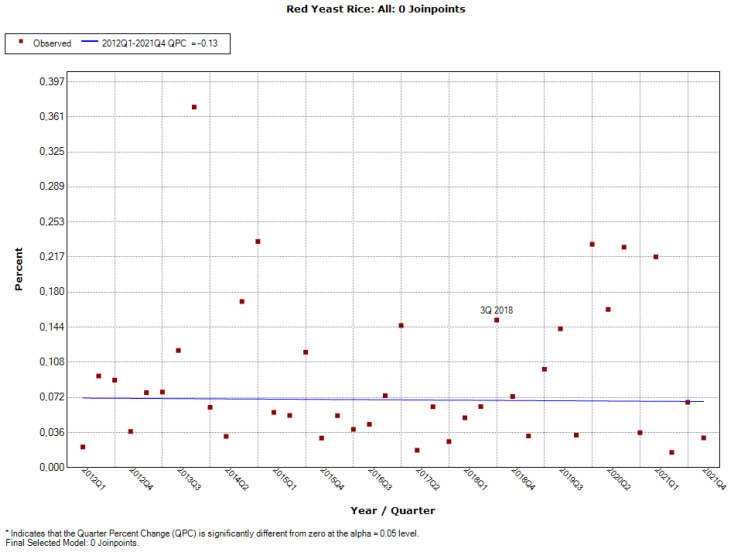
Percentage of products containing red yeast rice in individual quarters in the period from 2012 to 28 November 2021. Joinpoints mark trend turning points. The date above the observation indicates the moment of publication of the scientific opinion/resolution.

**Figure A15:**
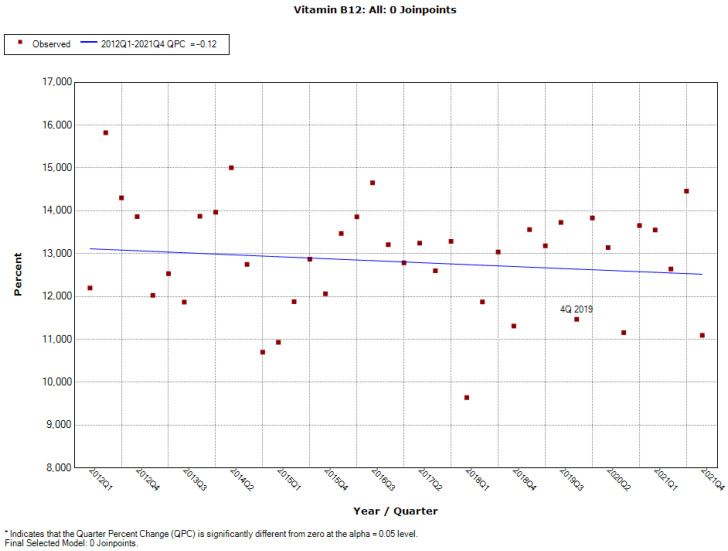
Percentage of products containing vitamin B12 in individual quarters in the period from 2012 to 28 November 2021. Joinpoints mark trend turning points. The date above the observation indicates the moment of publication of the scientific opinion/resolution.

**Figure A16:**
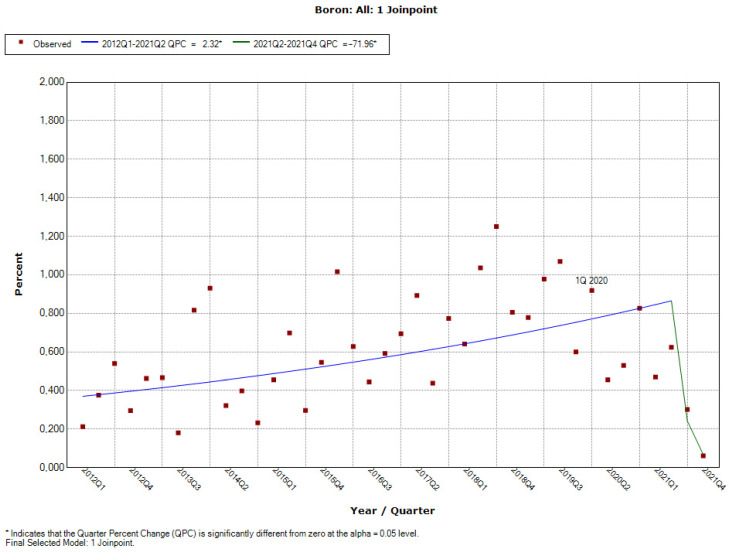
Percentage of products containing boron in individual quarters in the period from 2012 to 28 November 2021. Joinpoints mark trend turning points. The date above the observation indicates the moment of publication of the scientific opinion/resolution.

**Figure A17:**
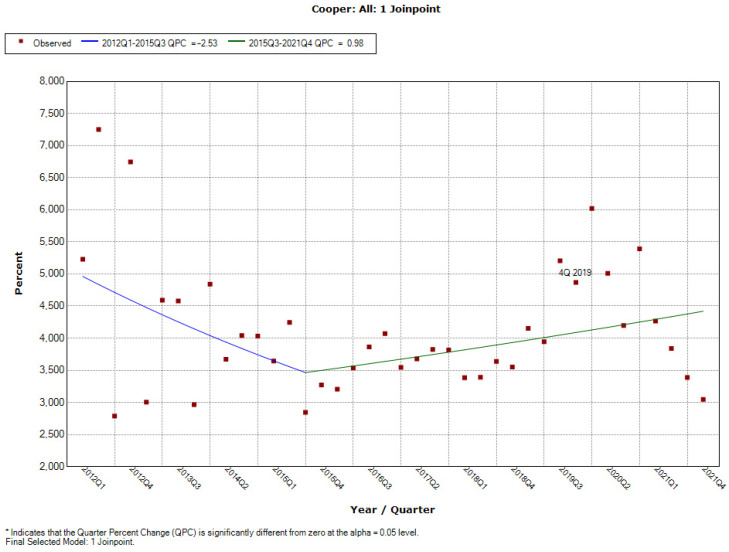
Percentage of products containing cooper in individual quarters in the period from 2012 to 28 November 2021. Joinpoints mark trend turning points. The date above the observation indicates the moment of publication of the scientific opinion/resolution.

**Figure A18:**
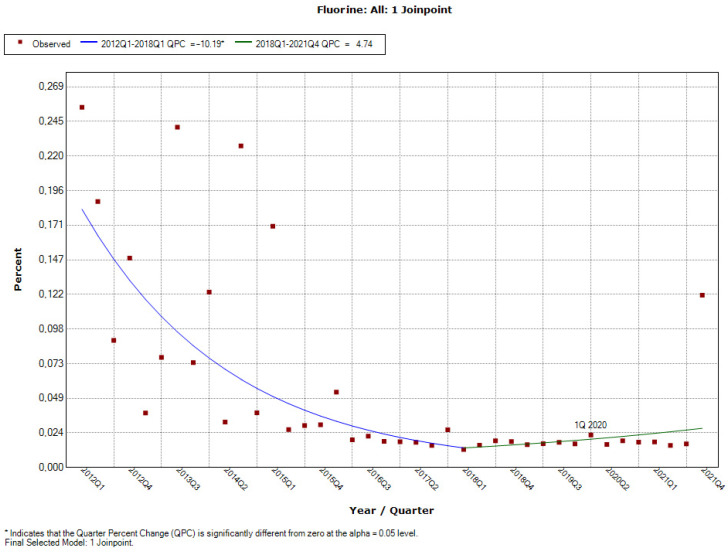
Percentage of products containing fluorine in individual quarters in the period from 2012 to 28 November 2021. Joinpoints mark trend turning points. The date above the observation indicates the moment of publication of the scientific opinion/resolution.

**Figure A19:**
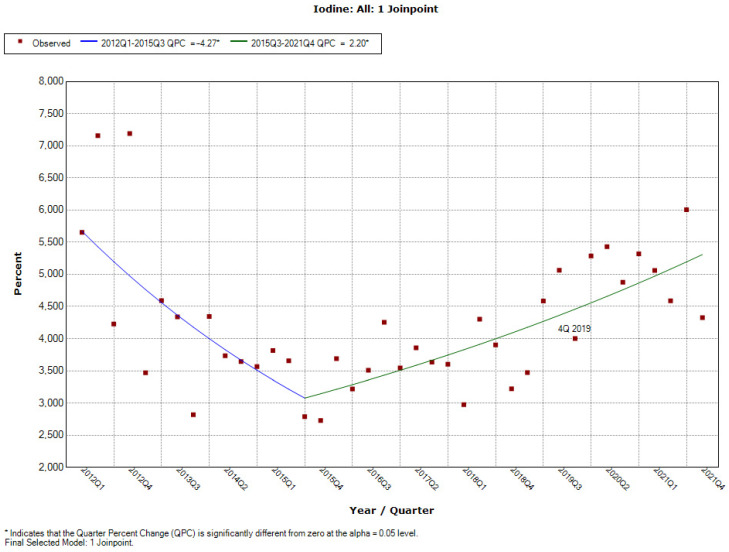
Percentage of products containing iodine in individual quarters in the period from 2012 to 28 November 2021. Joinpoints mark trend turning points. The date above the observation indicates the moment of publication of the scientific opinion/resolution.

**Figure A20:**
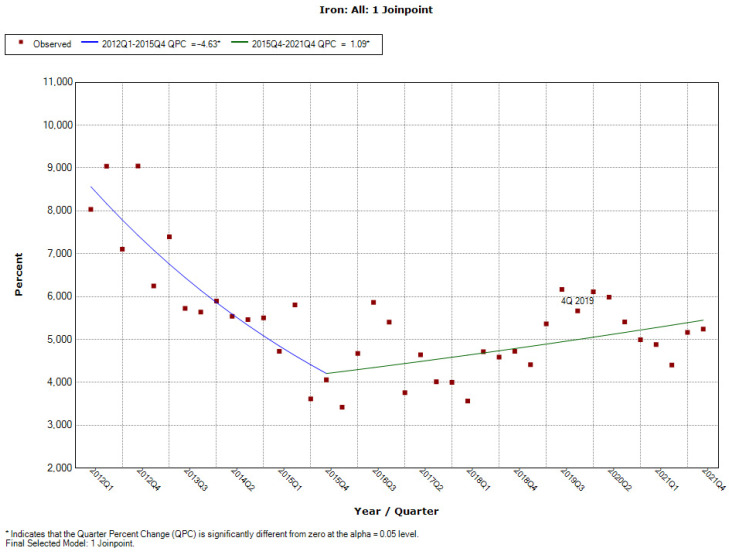
Percentage of products containing iron in individual quarters in the period from 2012 to 28 November 2021. Joinpoints mark trend turning points. The date above the observation indicates the moment of publication of the scientific opinion/resolution.

**Figure A21:**
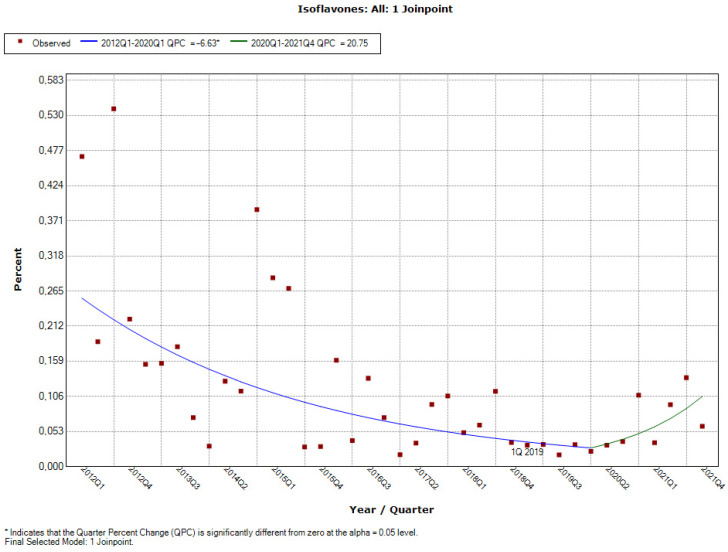
Percentage of products containing isoflavones in individual quarters in the period from 2012 to 28 November 2021. Joinpoints mark trend turning points. The date above the observation indicates the moment of publication of the scientific opinion/resolution.

**Figure A22:**
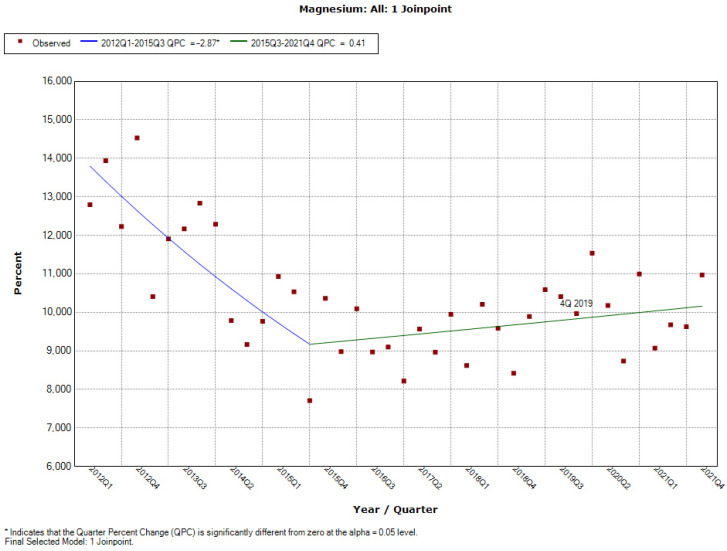
Percentage of products containing magnesium in individual quarters in the period from 2012 to 28 November 2021. Joinpoints mark trend turning points. The date above the observation indicates the moment of publication of the scientific opinion/resolution.

**Figure A23:**
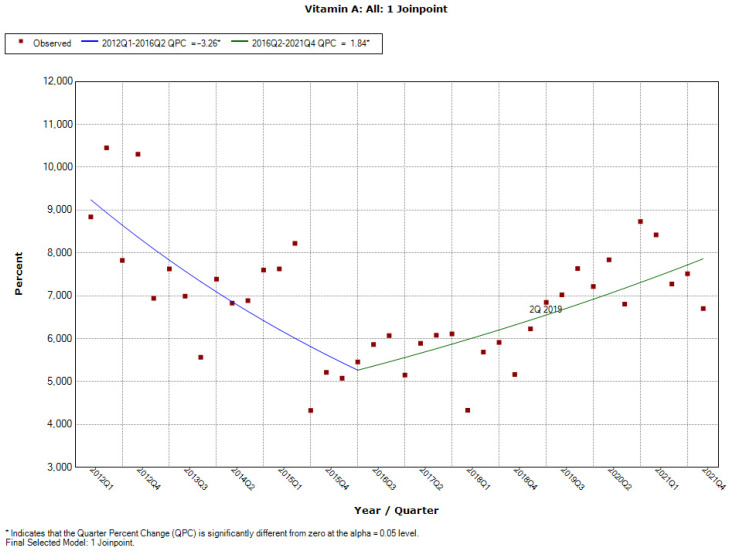
Percentage of products containing vitamin A in individual quarters in the period from 2012 to 28 November 2021. Joinpoints mark trend turning points. The date above the observation indicates the moment of publication of the scientific opinion/resolution.

**Figure A24:**
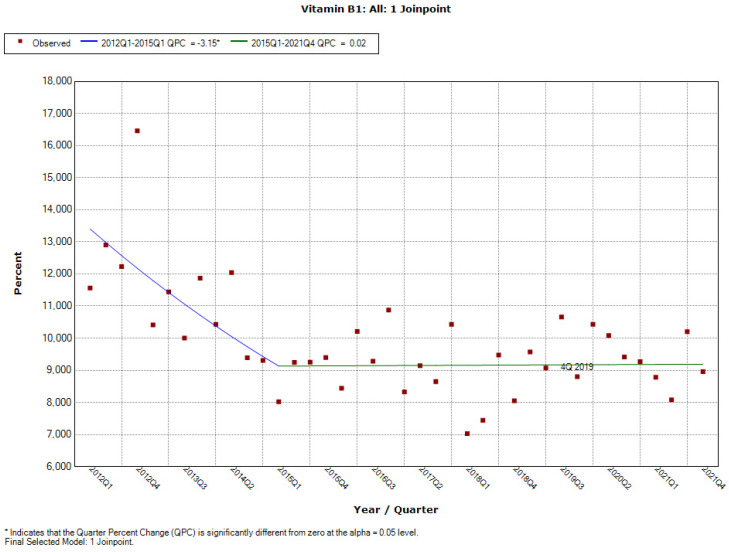
Percentage of products containing vitamin B1 in individual quarters in the period from 2012 to 28 November 2021. Joinpoints mark trend turning points. The date above the observation indicates the moment of publication of the scientific opinion/resolution.

**Figure A25:**
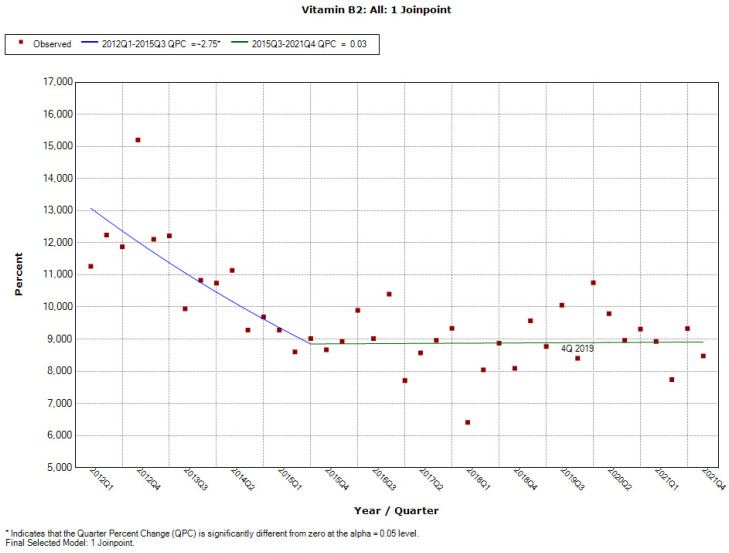
Percentage of products containing vitamin B2 in individual quarters in the period from 2012 to 28 November 2021. Joinpoints mark trend turning points. The date above the observation indicates the moment of publication of the scientific opinion/resolution.

**Figure A26:**
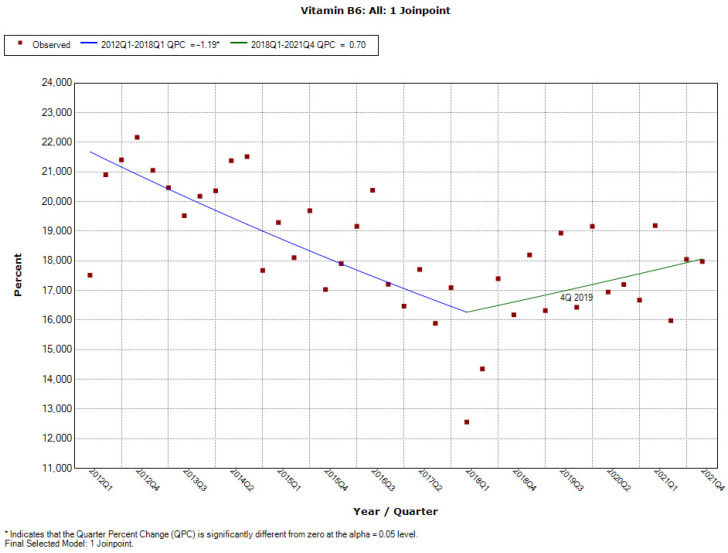
Percentage of products containing vitamin B6 in individual quarters in the period from 2012 to 28 November 2021. Joinpoints mark trend turning points. The date above the observation indicates the moment of publication of the scientific opinion/resolution.

**Figure A27:**
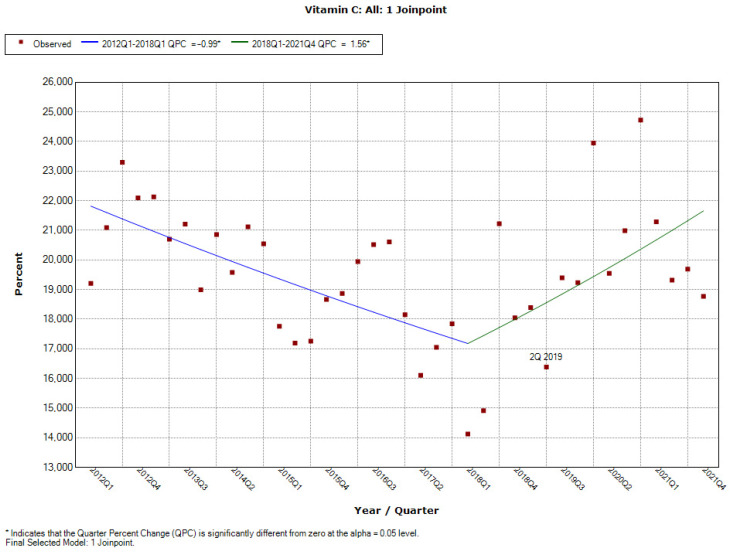
Percentage of products containing vitamin C in individual quarters in the period from 2012 to 28 November 2021. Joinpoints mark trend turning points. The date above the observation indicates the moment of publication of the scientific opinion/resolution.

**Figure A28:**
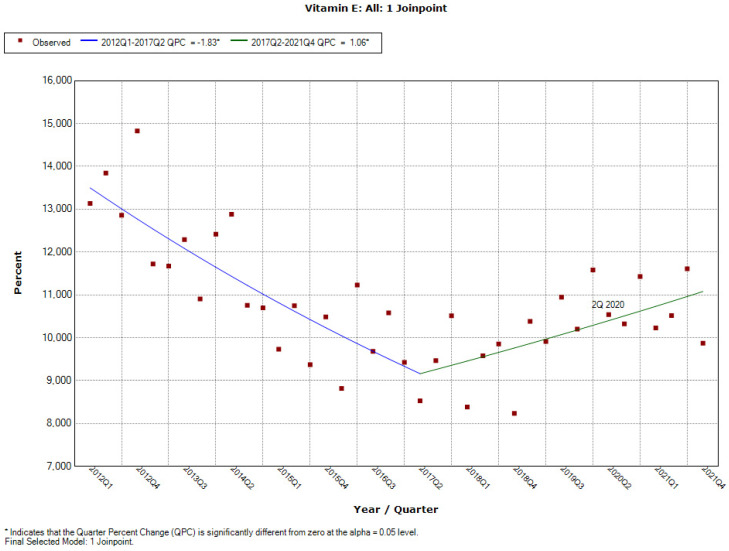
Percentage of products containing vitamin E in individual quarters in the period from 2012 to 28 November 2021. Joinpoints mark trend turning points. The date above the observation indicates the moment of publication of the scientific opinion/resolution.

**Figure A29:**
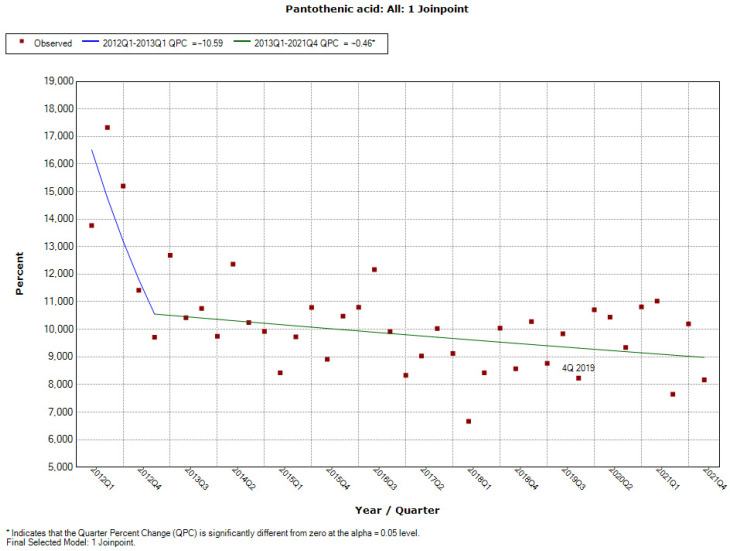
Percentage of products containing pantothenic acid in individual quarters in the period from 2012 to 28 November 2021. Joinpoints mark trend turning points. The date above the observation indicates the moment of publication of the scientific opinion/resolution.

**Figure A30:**
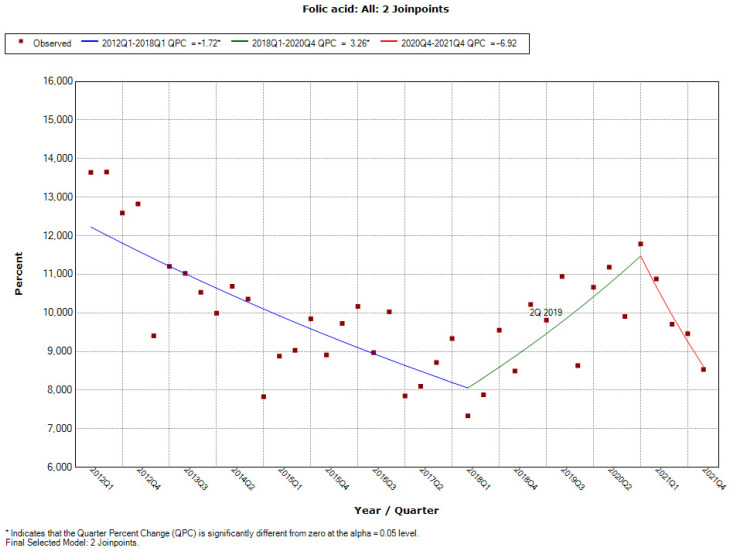
Percentage of products containing folic acid in individual quarters in the period from 2012 to 28 November 2021. Joinpoints mark trend turning points. The date above the observation indicates the moment of publication of the scientific opinion/resolution.

**Figure A31:**
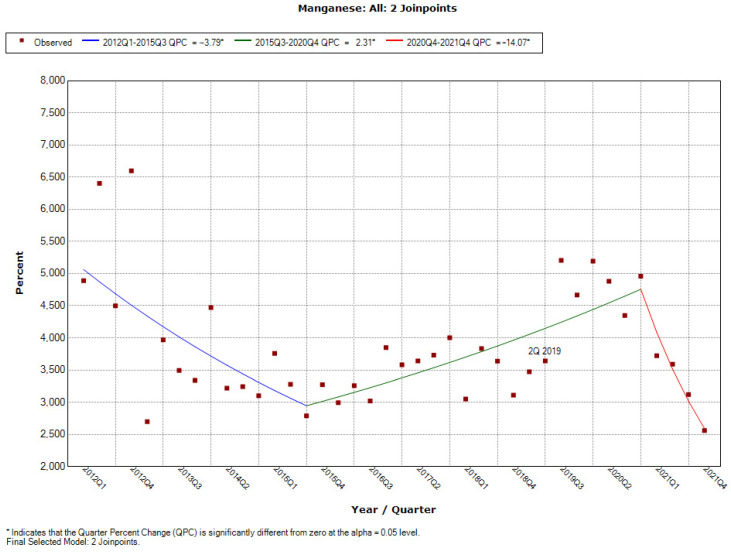
Percentage of products containing manganese in individual quarters in the period from 2012 to 28 November 2021. Joinpoints mark trend turning points. The date above the observation indicates the moment of publication of the scientific opinion/resolution.

**Figure A32:**
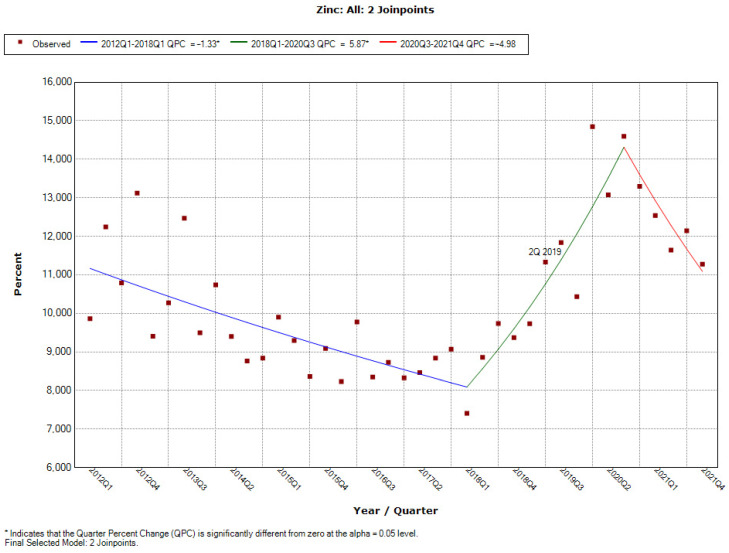
Percentage of products containing zinc in individual quarters in the period from 2012 to 28 November 2021. Joinpoints mark trend turning points. The date above the observation indicates the moment of publication of the scientific opinion/resolution.

**Figure A33:**
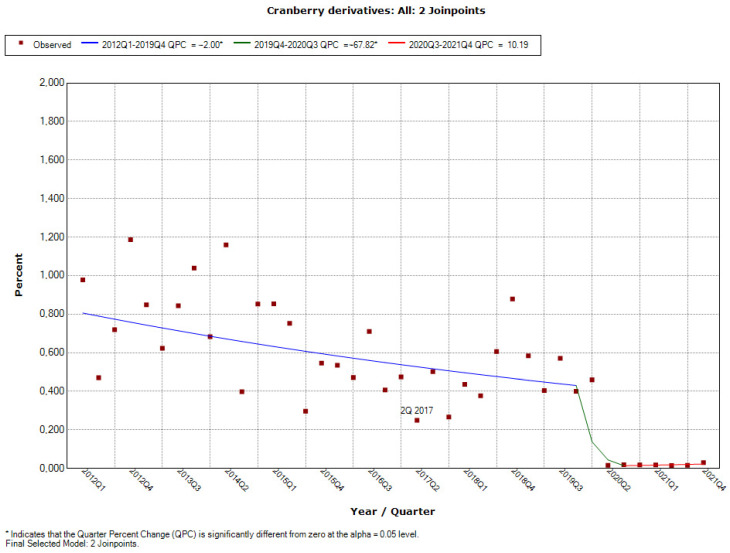
Percentage of products containing cranberry derivatives in individual quarters in the period from 2012 to 28 November 2021. Joinpoints mark trend turning points. The date above the observation indicates the moment of publication of the scientific opinion/resolution.

**Figure A34:**
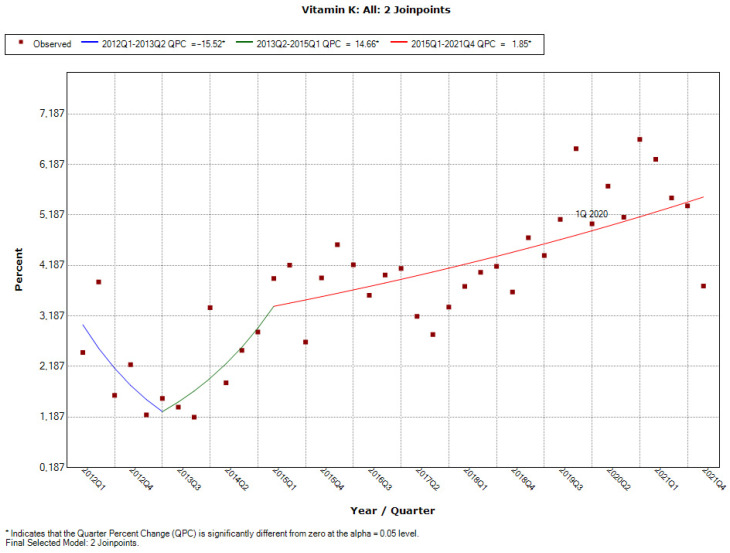
Percentage of products containing vitamin K in individual quarters in the period from 2012 to 28 November 2021. Joinpoints mark trend turning points. The date above the observation indicates the moment of publication of the scientific opinion/resolution.

**Figure A35:**
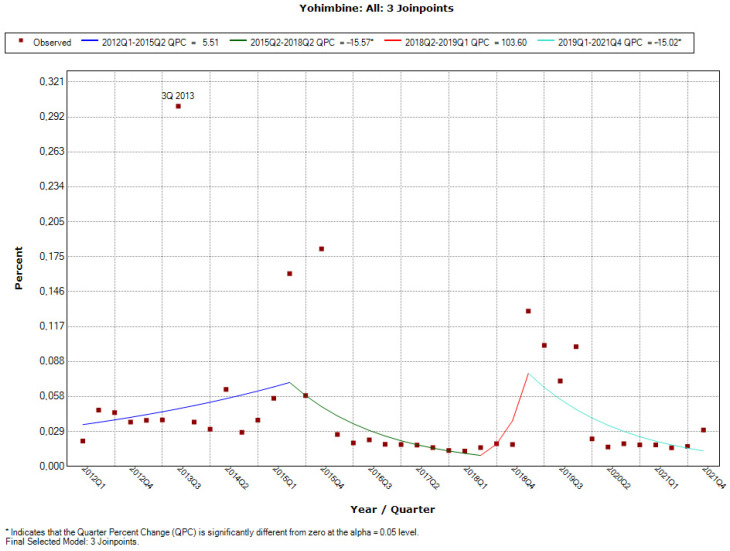
Percentage of products containing yohimbine in individual quarters in the period from 2012 to 28 November 2021. Joinpoints mark trend turning points. The date above the observation indicates the moment of publication of the scientific opinion/resolution.

**Figure A36:**
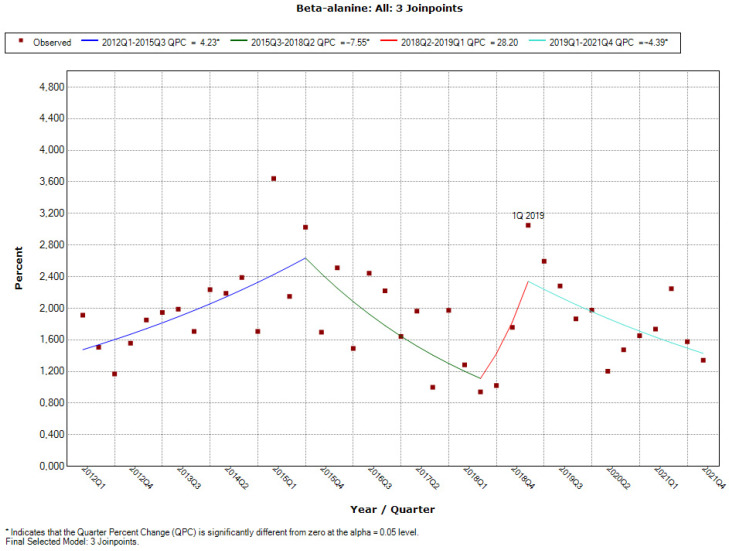
Percentage of products containing beta-alanine in individual quarters in the period from 2012 to 28 November 2021. Joinpoints mark trend turning points. The date above the observation indicates the moment of publication of the scientific opinion/resolution.

**Figure A37:**
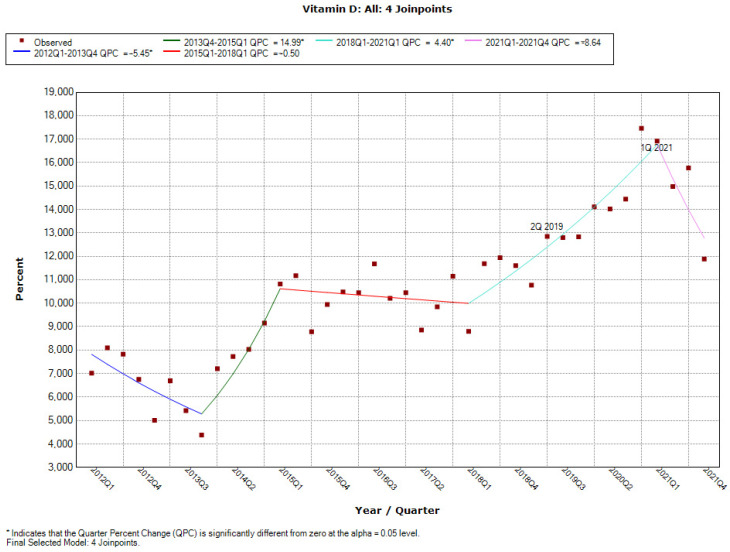
Percentage of products containing vitamin D in individual quarters in the period from 2012 to 28 November 2021. Joinpoints mark trend turning points. The date above the observation indicates the moment of publication of the scientific opinion/resolution.

## Data Availability

Publicly available datasets were analysed in this study. This data can be found here: https://powiadomienia.gis.gov.pl (accessed on 28 November 2021); https://www.gov.pl/web/gis/zespol-do-spraw-suplementow-diety (accessed on 7 November 2021).

## Appendix A

**Table A1:** Quality requirements and maximum levels of vitamins, minerals, plant ingredients and other ingredients with a physiological effect in dietary supplements.

Ingredient	Maximum Levels and Other Requirements	Resolution Number (Date)
Vitamin A	800 g of retinol equivalent (retinol and retinyl esters); 7 mg of β-carotene.	6/2019 (11 June 2019)
Vitamin D	2000 IU (50 g) for dietary supplements intended for a healthy adult population up to 75 years of age; 4000 IU (100 g) for dietary supplements intended only for healthy people over 75 years of age.	1/2021 (19 February2021)
Vitamin E	250 mg	1/2020 (7 February 2020)
Vitamin K	200 g	2/2020 (7 February 2020)
Vitamin C	1000 mg	5/2019 (11 June 2019)
Vitamin B1 (thiamine)	100 mg	12/2019 (25 October 2019)
Vitamin B2 (riboflavin)	40 mg	13/2019 (25 October 2019)
Niacin	830 mg as nicotinamide 16 mg as nicotinic acid	8/2019 (11 June 2019)
Vitamin B6	18 mg	18/2019 (13 December 2019)
Folic acid	600 g 800 g in supplements marked as dedicated to pregnant women	7/2019 (11 June 2019)
Vitamin B12	100 g	14/2019 (25 October 2019)
Pantothenic acid	10 mg of pantethine, 200 mg in other chemical forms as pantothenic acid	11/2019 (25 October 2019)
Boron	3 mg	3/2020 (7 February 2020)
Chromium	200 g	4/2020 (7 February 2020)
Zinc	15 mg	10/2019 (11 June 2019)
Fluorine	3.5 mg	5/2020 (7 February 2020)
Phosphorus	450 mg	6/2020 (7 February 2020)
Iodine	150 g> 200 g in dietary supplements marked as dedicated for pregnant and lactating women	15/2019 (25 October 2019)
Magnesium	400 mg	19/2019 (13 December 2019)
Manganese	1.8 mg	9/2019 (11 June 2019)
Copper	2 mg	21/2019 (13 December 2019)
Iron	20 mg 30 mg in supplements marked as dedicated to pregnant women	20/2019 (13 December 2019)
Isoflavones	Soy isoflavones can be used in an amount up to 100 mg/day, in divided portions (at least two); The remaining isoflavones (from other plant sources) can be used up to 50 mg/day.	1/2019 (14 January 2019)
Caffeine	Caffeine can be used in an amount up to 400 mg a day, in divided portions, provided that the product does not contain other ingredients with a synergistic effect; A portion of caffeine consumed at one time must not exceed 200 mg.	16/2019 (25 October 2019)
White mulberry (*Morus alba* L.)	The maximum content of 1-deoxynojirmycin (DNJ)-at the level of 10 mg in the recommended daily portion of the product; An entity placing a given food on the market should have information on the content of DNJ, per the recommended daily portion of the product.	17/2019 (25 October 2019)
Aloe	Only inner leaf gel/pulp may be used; Must not contain hydroxyanthracene compounds; Aloin A and B levels below 10 mg/kg (10 ppm); Cannot be used in products for children, pregnant and lactating women.	2/2019 (14 January 2019)
Ashwagandha (*Withania somnifera*)	*Withania somnifera* (L.) Dunal root powder can be used in an amount less than 3 g per day; The maximum content of withanolides cannot exceed 10 mg in the recommended daily portion of the product; An entity placing a given food on the market should attach a quantitative specification confirming the content of the sum of withanolides per the recommended daily portion of the product.	7/2020 (7 February 2020)
Beta-alanine	Beta-alanine can be used in the amount of up to 2400 mg/day in divided portions; A portion of beta-alanine consumed at one time cannot exceed 800 mg.	3/2019 (14 January 2019)
